# From Epithelial Sensing to Visceral Pain: Neuropod and Enterochromaffin Cells in Gut Neuroepithelial Circuits

**DOI:** 10.3390/ijms27115109

**Published:** 2026-06-04

**Authors:** Agnieszka Nowacka, Maciej Śniegocki, Ewa A. Ziółkowska

**Affiliations:** 1 Department of Neurosurgery, Collegium Medicum in Bydgoszcz, Nicolaus Copernicus University in Toruń, ul. Curie Skłodowskiej 9, 85-094 Bydgoszcz, Poland; 2 Department of Animal Biotechnology and Genetics, Faculty of Animal, Breeding and Biology, Bydgoszcz University of Science and Technology, Mazowiecka 28, 85-084 Bydgoszcz, Poland

**Keywords:** visceral pain, neuroepithelial circuits, neuropod cells, enterochromaffin cells, gut–brain communication, GUCY2C, visceral hypersensitivity, intestinal sensory signaling

## Abstract

Visceral pain is a central feature of chronic gastrointestinal disorders, yet the epithelial sensory mechanisms that shape afferent input before it enters pain-relevant neural pathways remain insufficiently integrated into current models. This review advances the concept that the intestinal epithelium is not only a barrier or endocrine interface, but also an active neuroepithelial regulatory layer positioned upstream of visceral sensory signaling. Neuropod-cell studies established that specialized epithelial cells can communicate rapidly with vagal neurons and preserve luminal stimulus identity through transmitter-selective coding. Enterochromaffin cells extend this framework as polymodal epithelial sensory transducers that detect chemical, microbial, neurohumoral, and mechanical cues, convert them into serotonergic afferent signaling, and can causally amplify visceral hypersensitivity in experimental models. Complementing these amplifying pathways, GUCY2C^high^ (guanylate cyclase C-enriched) neuropod-like epithelial cells reveal a pain-restraining mechanism that regulates dorsal root ganglion excitability and preserves linaclotide-responsive suppression of nociceptive output in preclinical systems. Together, these findings support an integrative model in which epithelial sensory circuits may act as filters of biological meaning, amplifiers of afferent gain, and brakes on aberrant nociceptive escalation. This framework does not replace neural, immune, or central mechanisms of visceral pain, but adds an upstream epithelial tier that may shape pain vulnerability, persistence, or treatment responsiveness in selected contexts. Defining the cellular logic, molecular mediators, and human relevance of these circuits will be essential for advancing neuroepithelial pain biology toward disease-relevant and therapeutic applications.

## 1. Introduction

Visceral pain remains one of the most clinically consequential and therapeutically difficult manifestations of chronic gastrointestinal dysfunction [1,2,3]. It is a defining feature of disorders of gut–brain interaction, particularly irritable bowel syndrome (IBS), and also contributes substantially to persistent symptom burden in inflammatory bowel conditions [4,5,6,7]. Unlike transient protective nociception, chronic visceral pain may become disproportionate to ongoing tissue injury, poorly responsive to available treatments, and deeply disruptive to quality of life. This challenge has created a persistent need for more effective, mechanism-based, non-opioid therapeutic strategies and for a clearer understanding of the biological processes that initiate and sustain pain-related signaling in the gut [7,8,9,10]. Progress will therefore require not only improved approaches to dampening established hypersensitivity, but also a more precise account of how intestinal sensory information is organized before it enters pain-relevant neural pathways.

Contemporary models of visceral pain have rightly emphasized sensitization of peripheral nociceptors, neuroimmune crosstalk, altered epithelial barrier function, and amplification within spinal, supraspinal, and brain–gut processing networks [11,12,13,14]. These mechanisms remain indispensable for understanding why visceral afferent pathways become hyperexcitable, why pain may persist after an inciting insult, and how local intestinal signals are transformed into chronic symptom states. Yet these frameworks have largely focused on how pain pathways become sensitized, rather than on how biologically meaningful luminal information is initially converted into afferent signals capable of entering those pathways [12,15,16]. The unresolved issue is not whether neural sensitization matters, but whether the field has sufficiently defined the epithelial events that determine what reaches sensitized pathways in the first place. In this sense, a key conceptual question lies upstream of the nerve terminal: what sensory mechanisms at the mucosal interface determine whether intestinal signals are ignored, physiologically encoded, or admitted into nociceptive circuits?

A growing body of work now suggests that this upstream sensory step may reside, at least in part, within the intestinal epithelium itself [17,18,19]. The gut epithelium is increasingly difficult to view only as a physical barrier, secretory surface, or inflammatory interface. Rather, it contains rare specialized sensory cell populations capable of engaging neural pathways with a degree of structural and functional specificity that challenges older diffusible-signaling models. The discovery that sensory enteroendocrine cells can participate in organized neuroepithelial circuits established that the epithelial layer possesses direct nerve-oriented signaling capacity [2,20,21]. Subsequent work showed that specialized epithelial sensory cells can transfer luminal information into neural circuits with rapid functional precision, demonstrating that epithelial-to-neural communication can operate with the temporal features expected of a sensory system [22]. In parallel, enterochromaffin cells emerged as polymodal epithelial sensory transducers capable of detecting biologically salient intestinal cues and coupling their responses to primary sensory pathways [23]. Taken together, these findings indicate that the epithelium itself may participate in neural information processing. Although the foundational studies establishing neuroepithelial communication were not designed as direct models of chronic visceral pain, they opened a new conceptual space in which epithelial sensory cells could operate upstream of nociceptive escalation, shaping whether intestinal signals are transmitted, amplified, restrained, or functionally filtered. More recent work linking specialized epithelial signaling states to visceral pain regulation further underscores the relevance of this emerging framework [10].

These discoveries have transformed epithelial sensory biology, but their implications for visceral pain have not yet been fully integrated into a coherent conceptual model. This review examines how discoveries in neuropod-cell biology, enterochromaffin-cell sensory transduction, epithelial–afferent communication, and guanylate cyclase C (GUCY2C)-dependent analgesic signaling converge on a broader concept: the gut epithelium as an active neuroepithelial regulator of visceral pain. The aim is not to replace neuron-centered models of visceral nociception, neuroimmune amplification, or central sensitization, but to complete them by incorporating the epithelial sensory layer that precedes and shapes afferent activation. In this framework, specialized epithelial cells may contribute to the initiation of pain-relevant signaling, the amplification of afferent gain, the classification of biologically meaningful intestinal stimuli, and the restraint of aberrant nociceptive output.

## 2. The Gut Epithelium as a Sensory Neural Interface: From Endocrine Signaling to Neuroepithelial Communication

### 2.1. Classical Enteroendocrine Signaling: Endocrine and Paracrine Regulation of Luminal Information

Enteroendocrine cells (EECs) have traditionally been viewed as sparse, specialized epithelial sensors that translate luminal information into coordinated physiological responses [1,24,25]. Distributed throughout the intestinal mucosa, they detect nutrients and other gut-derived chemical cues and respond by releasing bioactive peptides and hormones [24,26,27]. In the classical model, these signals act mainly through endocrine secretion into the circulation or paracrine communication within the local mucosal environment. Through these diffusible routes, enteroendocrine cells regulate nutrient handling, gastric emptying, intestinal secretion and motility, pancreatic endocrine responses, satiety, and feeding-related physiology [1,24,28,29].

This framework established enteroendocrine cells as active sensory elements rather than passive hormone reservoirs. However, it also reinforced a simplified view of them as dispersed epithelial secretory units acting primarily through molecules released into extracellular or vascular spaces. Their sparse distribution and the historical reliance on peptide immunolabeling further supported the image of relatively compact flask- or spindle-shaped cells embedded within the epithelium [29,30,31].

Although endocrine and paracrine signaling remain central to enteroendocrine biology, they do not readily explain how luminal information could be transmitted to neural circuits with high spatial precision. Diffusible signaling is well suited for broad physiological coordination, but less so for directed communication with specific neural partners in the mucosa [32,33,34]. This limitation opened the way for a revised view of enteroendocrine cells, in which cell polarity, basal specializations, and structurally organized epithelial–neural communication became increasingly important.

### 2.2. From Basal Cytoplasmic Processes to Neuropods: Anatomical Evidence for Epithelial Neural Specialization

The first challenge to a purely diffusible view of enteroendocrine communication came from renewed attention to cell morphology. Bohórquez and Liddle [29] noted that some enteroendocrine cells extend prominent basal cytoplasmic processes beneath neighboring enterocytes and into the lamina propria. These projections can exceed 50 μm, resemble axons, and terminate in bouton-like enlargements. Although this observation did not establish a neural circuit, it suggested that some EECs may be structurally adapted for localized, directionally organized communication beneath the epithelium rather than relying only on diffuse endocrine or paracrine signaling [29].

This possibility gained strong anatomical support in 2014, when Bohórquez and colleagues reconstructed an enteroendocrine cell in three dimensions using correlative confocal microscopy and serial block-face scanning electron microscopy [35]. They identified the basal projection as a prominent, highly organized structure and termed it a neuropod. Approximately 73.5% of peptide-containing secretory vesicles in the reconstructed cell were located within the neuropod, with many concentrated near its terminal region [35]. This polarized vesicle distribution indicated that the basal projection may function as a specialized signaling compartment rather than as a passive morphological extension [35].

Neuropods also displayed neuron-like structural features. Thin filamentous elements interpreted as neurofilaments extended along the basal process, supporting the idea of a highly polarized cytoskeletal organization consistent with its axon-like appearance [35,36,37,38]. Importantly, this does not mean that enteroendocrine cells are neurons. Rather, it shows that a subset of epithelial sensory cells possesses cellular architecture compatible with targeted local communication.

A further notable finding was the close association between neuropods and enteric glial processes. Serial electron microscopy showed that glial extensions accompanied neuropods as they projected into the lamina propria [35]. In organoid experiments, glial-derived neurotrophic factors enhanced neuropod elaboration in vitro [35]. These observations suggest that neuropod development may be shaped, at least in part, by the local neural–glial microenvironment. Although this glial association did not demonstrate a mature neuronal circuit, it placed neuropods within a broader subepithelial signaling landscape.

Together, these findings marked a major conceptual shift. Some enteroendocrine cells are not only secretory epithelial sensors, but morphologically polarized cells with vesicle-rich basal projections, neuron-like cytoskeletal features, and developmental sensitivity to local glial cues [29,35]. These data did not yet establish direct neural communication, but they made a purely diffuse endocrine/paracrine model insufficient. The next question was therefore whether these anatomically specialized neuropods provide the structural basis for direct communication with intestinal nerves.

### 2.3. From Neuropod Morphology to Neuroepithelial Circuits: Molecular and Cellular Evidence for Direct Nerve-Oriented Communication

The anatomical definition of the neuropod raised a key question: were these basal processes simply unusual epithelial specializations, or did they participate in organized communication with nerves? Bohórquez et al. [21] addressed this in 2015, transforming the neuropod from a morphological observation into the basis of a new conceptual framework—the neuroepithelial circuit.

A central finding was that neuropod-bearing sensory enteroendocrine cells were oriented toward mucosal nerve fibers rather than the vasculature. While blood vessels were nearby, they did not directly contact enteroendocrine cells in a manner consistent with preferential endocrine export. In contrast, mucosal nerve fibers penetrated the basal lamina and directly contacted enteroendocrine neuropods in both the small intestine and colon [21]. This shifted the interpretation of EEC biology: at least a subset of these cells appeared structurally aligned with neural partners, not only with interstitial or vascular space [1,2,20,32].

This anatomical evidence was supported by molecular profiling. Enteroendocrine cells expressed genes encoding presynaptic, postsynaptic, and transsynaptic components, indicating machinery compatible with organized cell–cell communication beyond diffuse hormone release [21]. These markers do not by themselves prove mature functional synapses or imply that EECs are neurons [21]. However, together with their polarized morphology and direct nerve contacts, they provided strong evidence that certain epithelial sensory cells are equipped for nerve-oriented interaction [21,29,35].

Bohórquez et al. [21] also showed that enteroendocrine cells can form stable associations with sensory neurons in vitro. In coculture, epithelial and neuronal processes extended toward one another and established persistent contacts lasting several hours [21]. These observations argued against incidental tissue proximity and supported an intrinsic capacity for directed epithelial–neuronal interaction.

The study further strengthened this model using in vivo monosynaptic rabies tracing, which demonstrated that sensory enteroendocrine cells participate in a traceable epithelial-to-neural circuit within the intestinal mucosa [21]. This provided critical in vivo evidence that these cells are embedded in a neural communication route rather than merely positioned near nerves.

The significance of Bohórquez et al. [21] lies not in demonstrating rapid neurotransmission or pain signaling, but in redefining a subset of enteroendocrine cells as structured interfaces between luminal stimuli and the nervous system. These findings established the anatomical, molecular, and conceptual basis of neuroepithelial communication [21,29,35]. They also set up the next question: whether neuropod-bearing epithelial cells can transmit biologically meaningful luminal signals into defined neural circuits with the speed and specificity expected of a sensory system.

### 2.4. Terminological Clarification: Enteroendocrine Cells, Enterochromaffin Cells, Neuropod Cells, and Neuropod-like Cells

Because several epithelial sensory cell terms are used throughout this review, a brief terminological clarification is necessary. Enteroendocrine cells (EECs) represent the broad epithelial endocrine lineage that includes multiple hormone-producing cell types distributed along the gastrointestinal mucosa [1,24,26]. Enterochromaffin (EC) cells are a specialized serotonergic subset of EECs, defined primarily by TPH1-dependent serotonin/5-hydroxytryptamine (5-HT)production and release, and should not be treated as synonymous with all EECs [23]. Neuropod cells refer here to epithelial sensory cells, usually within the enteroendocrine lineage, that possess basal nerve-oriented processes and, where functionally demonstrated, communicate rapidly with defined neural partners [21,22,29,35]. In contrast, the term neuropod-like cell is used more cautiously for epithelial populations that show neuropod-associated morphology and molecular features, such as basal pseudopod-like projections or synaptic gene enrichment, but whose complete identity and transmission mode may not be identical to previously defined nutrient-sensing neuropod cells [10]. Thus, CCK-labeled nutrient-sensing neuropod cells, EC cells, and GUCY2C^high^ neuropod-like cells are related within the broader framework of epithelial sensory biology, but they represent distinct or only partially overlapping epithelial populations with different transmitter outputs, neural partners, and relevance to visceral pain.

## 3. Neuropod Cells and the Establishment of Rapid Gut–Brain Sensory Circuits

### 3.1. From Neuroepithelial Architecture to Rapid Sensory Transmission

The anatomical evidence for neuropod-bearing enteroendocrine cells raised a key functional question: can these epithelial cells transmit luminal information rapidly and specifically into neural circuits, or are they primarily secretory cells positioned near nerves? Kaelberer et al. [22] addressed this question by showing that neuropod cells can relay intestinal sensory information to vagal neurons with the speed and transmitter dependence expected of a neural signaling pathway. Their work marked the transition from anatomical evidence of epithelial–neuronal connectivity to the functional demonstration of a rapid gut–brain sensory circuit.

### 3.2. Rapid Synaptic Gut–Brain Transmission: Neuropod Cells as Glutamatergic Epithelial Sensory Transducers

The discovery of direct epithelial–neuronal contacts raised an important physiological question. Classical enteroendocrine peptides can strongly influence vagal and central pathways, but endocrine signaling is generally too slow to explain rapid neural responses to newly encountered luminal stimuli [1,31,39,40]. Kaelberer et al. [22] therefore asked whether neuropod cells provide a fast route for relaying intestinal sensory information to the brain, moving the field from anatomical description to functional sensory transduction.

Using circuit tracing, organoid–neuron cocultures, optogenetics, electrophysiology, and neurotransmitter reporters, they showed that enteroendocrine-derived neuropod cells form direct synaptic connections with vagal nodose neurons [22]. Monosynaptic rabies tracing further linked this epithelial–neural circuit to vagal sensory ganglia, establishing a one-synapse route from the intestinal mucosa toward the brainstem [22]. This finding defined a specific vagal gut–brain circuit; it should not be generalized to all epithelial–neuronal communication in the gastrointestinal tract or interpreted as a spinal nociceptive pathway.

The functional relevance of this circuit was demonstrated in glucose-sensing experiments. Isolated nodose neurons did not respond directly to glucose, whereas glucose stimulation evoked excitatory postsynaptic currents when nodose neurons were cocultured with intestinal epithelial sensory cells [22]. In vivo, luminal sugar increased vagal firing, optical silencing of enteroendocrine cells suppressed this response, and optogenetic activation of these cells was sufficient to drive vagal activity [22]. These findings placed neuropod cells upstream of vagal sensory signaling and identified them as epithelial transducers of luminal nutrient information in this experimental setting.

A major advance was the demonstration of millisecond-scale epithelial-to-neural signaling. Optogenetic activation of neuropod cells elicited rapid postsynaptic responses in connected vagal neurons, and analyses using the intensity-based glutamate-sensing fluorescent reporter (iGluSnFR) identified glutamate as the fast transmitter mediating sugar-evoked signaling to vagal afferents [22]. Thus, the gut epithelium can communicate with sensory neural pathways not only through slower endocrine outputs, but also through rapid transmitter-based signaling [22].

### 3.3. Neuropod Cells Encode Luminal Identity Through Transmitter-Selective Output

Buchanan et al. [41] extended neuropod-cell biology from rapid sensory transmission to sensory coding. They investigated how animals distinguish nutritive sugars from non-caloric sweeteners despite similar oral sweetness, a behavior known to depend in part on postingestive intestinal signals [42,43,44,45]. Their study positioned CCK-labeled duodenal neuropod cells as epithelial intermediaries in this discrimination.

Intraduodenal delivery of several nutritive sugars and artificial sweeteners elicited rapid vagal responses, whereas fructose did not under the conditions tested [41]. These responses were specific to the proximal small intestine and required an epithelial intermediary, as isolated nodose neurons failed to respond directly to glucose, maltodextrin, or sucralose [41]. Optical silencing of CCK-labeled duodenal neuropod cells abolished vagal responses to sucrose, α-methylglucopyranoside, and sucralose, while coculture experiments showed stimulus-evoked postsynaptic currents only when nodose neurons were paired with neuropod cells [41]. These data identify duodenal neuropod cells as necessary intermediaries for rapid vagal responses to both nutritive and non-nutritive sweet stimuli in this circuit.

The key advance was that neuropod cells did not merely detect sweet luminal cues; they distinguished between them. CCK-labeled neuropod cells expressed partially overlapping receptor programs, including *Slc5a1*, encoding sodium-glucose cotransporter 1 (SGLT1), and *Tas1r3*, encoding taste receptor type 1 member 3 (T1R3) [41]. The data supported a model in which nutritive sugars are detected mainly through SGLT1-dependent uptake, whereas sweetener responses involve T1R3-associated sensing [46,47]. The near absence of *Tas1r2* transcripts is an important nuance, indicating that this mechanism should not be equated with canonical tongue-like taste receptor type 1 members 2 and 3 (T1R2/T1R3) taste signaling [41,46,47]. Instead, the study revealed a gut-specific sensory logic for parsing related luminal stimuli.

This discrimination was also reflected in transmitter output. In the duodenal CCK-labeled neuropod-cell system, nutritive sugar signaling relied on glutamatergic transmission, whereas sweetener signaling engaged purinergic transmission [41]. Neuropod cells therefore preserve information about stimulus identity and route it into distinct neural outputs. Disruption of glutamatergic neuropod-cell signaling altered preference for nutritive sugar over sweetener, showing that this epithelial code has behavioral relevance [41]. The study did not address pain, aversion, or nociception directly. Its importance for this review is that specialized epithelial sensory cells can shape not only whether luminal information reaches the nervous system, but also how that information is represented neurally.

### 3.4. Why Neuropod-Cell Sensory Biology Matters for Visceral Pain

The foundational neuropod-cell studies were not designed to test nociception, visceral hypersensitivity, or chronic pain. Bohórquez et al. [21,29,35] established epithelial–neuronal connectivity, Kaelberer et al. [22] defined a rapid vagal nutrient-sensing circuit, and Buchanan et al. [41] showed stimulus-selective epithelial coding linked to sugar preference. None of these studies directly demonstrated that neuropod cells generate or modulate pain signals.

Their relevance to visceral pain is therefore conceptual rather than direct. Together, these studies show that specialized epithelial cells can form directed neural contacts, communicate rapidly through classical neurotransmitters, engage vagal extrinsic sensory pathways, discriminate related luminal stimuli, and produce behaviorally meaningful neural outputs [21,22,29,35,41]. These features establish an epithelial sensory logic in which gut-derived information can be selected, transformed, and routed before it enters neural circuits.

This inference must remain anatomically bounded. Kaelberer et al. [22] and Buchanan et al. [41] primarily examined vagal gut–brain circuits, whereas visceral nociception is more often linked to spinal extrinsic afferents and dorsal root ganglion neurons. These pathways should not be treated as interchangeable. Instead, the neuropod literature provides a mechanistic precedent: the intestinal epithelium can act as a rapid sensory neural interface, but pain-focused studies must determine whether related principles extend to nociceptive circuits.

This transition becomes clearer in enterochromaffin cells, which detect irritants, microbial metabolites, catecholaminergic signals, and mechanical force, and convert these inputs into serotonergic communication with sensory afferents. Later sections will show that epithelial activity can recruit pain-relevant afferent pathways and that GUCY2C-dependent neuropod-like signaling regulates visceral nociceptive output. Thus, the main contribution of early neuropod-cell biology to visceral pain research is not direct evidence of nociception, but the discovery of a neuroepithelial sensory logic that later work has begun to extend into afferent excitability and analgesic regulation.

## 4. Enterochromaffin Cells as Polymodal Epithelial Sensory Transducers

### 4.1. Enterochromaffin Cells Are Electrically Excitable Epithelial Sensory Cells

The neuropod-cell literature established that the intestinal epithelium can engage neural circuits through rapid, transmitter-based signaling [1,3,10,48,49]. Enterochromaffin (EC) cells represent a distinct but complementary epithelial sensory system [1,3,50,51]. Although they have long been recognized as the major source of peripheral serotonin in the gastrointestinal tract [52,53], they are now understood as specialized sensory cells that detect biologically relevant changes in the gut environment and convert them into regulated secretory outputs [1,3,23,27,54,55]. Bellono et al. [23] provided key mechanistic evidence for this view by directly examining EC-cell excitability, receptor logic, and neural coupling in genetically labeled murine intestinal organoids.

A central feature of sensory transduction is the ability to convert receptor activation into changes in membrane excitability. Bellono et al. [23] showed that EC cells possess functional voltage-gated Na^+^ and Ca^2+^ conductances, generate action potentials, and, in a subset of cells, exhibit spontaneous Ca^2+^ bursts. These responses were sensitive to tetrodotoxin and P/Q-type Ca^2+^ channel inhibition, while transcriptomic data confirmed enrichment of ion channel genes consistent with electrical excitability, including *Scn3a* and voltage-gated calcium channel (CaV) components [23]. EC cells therefore possess the electrophysiological machinery required to act as active epithelial sensory transducers rather than passive serotonin reservoirs. Importantly, excitability alone does not imply nociception; it provides the biophysical basis through which gut-derived signals can be rapidly encoded and converted into regulated output [23].

The next question is therefore which biologically meaningful stimuli EC cells are equipped to detect, a point that becomes central to their later relevance for visceral pain biology.

### 4.2. Chemical Input Coding in EC Cells: Irritants, Microbial Metabolites, and Catecholaminergic Stress Signals

An early mechanistic entry point into EC-cell chemosensation came from Nozawa et al. [56], who identified the irritant-sensitive ion channel transient receptor potential ankyrin 1 (TRPA1) as a candidate sensory molecule in EC cells. TRPA1 mRNA was enriched in EC-cell-containing intestinal fractions and detected in 5-HT-positive epithelial cells in rat and human duodenum. Functionally, TRPA1 agonists, including allyl isothiocyanate and cinnamaldehyde, increased intracellular Ca^2+^ and stimulated 5-HT release in EC-enriched preparations and EC-like model cells [56]. In isolated guinea-pig ileum, allyl isothiocyanate enhanced contractility through a 5-HT_3_ receptor-dependent mechanism. Although this work did not establish direct EC-cell coupling to primary afferents or test pain, it showed that EC cells possess receptor-defined chemosensory machinery capable of translating luminal irritant cues into serotonergic physiological output [56].

Bellono et al. [23] substantially broadened this framework by showing that EC cells are polymodal chemosensors rather than responders to a single irritant class. They identified three major stimulus groups that activate EC cells through separable molecular pathways: reactive irritants, microbial metabolites, and catecholaminergic stress-related mediators [23]. This distinction is important because it shows that EC cells are not simply activated by nonspecific epithelial stress, but can classify different perturbations in the gut microenvironment.

The irritant-sensing branch involved TRPA1. Bellono et al. [23] confirmed robust EC-cell responses to allyl isothiocyanate and related electrophilic TRPA1 agonists, which were suppressed by TRPA1 antagonism, thereby extending the earlier observations of Nozawa et al. [56]. These data do not show that TRPA1-dependent EC-cell activation directly causes visceral pain, but they define a molecular route through which noxious luminal cues can be rapidly detected at the epithelial surface [23,56].

A second pathway concerns microbial metabolite sensing. Bellono et al. [23] identified Olfr558 as a mediator of EC-cell responses to isovalerate, a branched-chain fatty acid linked to microbial fermentation. Isovalerate evoked Ca^2+^ responses in EC cells, and CRISPR-mediated disruption of *Olfr558* abolished these responses while preserving sensitivity to unrelated stimuli such as allyl isothiocyanate (AITC) [23]. This genetic specificity supports the view that microbial metabolites are sensed through defined EC-cell receptor mechanisms rather than acting as nonspecific irritants. The resulting microbial metabolite–EC-cell axis is mechanistically important because it provides a pathway that can later be linked to pain-relevant sensory signaling [23].

A third input pathway connects EC cells to host stress physiology. Bellono et al. [23] showed that epinephrine and norepinephrine activate EC cells through an Adra2A–TRPC4-dependent cascade, with basolateral Adra2A localization consistent with detection of host-derived neurohumoral signals rather than luminal catecholamines. These findings position EC cells as epithelial interpreters of internal physiological state as well as environmental and microbial cues. However, they should not be taken as direct evidence that this pathway explains stress-exacerbated abdominal pain; rather, they identify a plausible cellular interface through which catecholaminergic tone may modulate EC-cell excitability and downstream sensory signaling [23].

Together, TRPA1-dependent irritant sensing, Olfr558-dependent microbial metabolite detection, and Adra2A–TRPC4-dependent catecholamine signaling define EC cells as chemical input coders rather than indiscriminate responders to epithelial disturbance. These pathways represent distinct sensory modalities that converge on EC-cell excitability and regulated secretory output [23,56]. This receptor-level diversity is central to the emerging view of EC cells as polymodal epithelial sensory transducers relevant to gastrointestinal discomfort and later pain-focused signaling.

### 4.3. Serotonin-Dependent Coupling of EC-Cell Activation to Sensory Afferent Pathways

The identification of receptor-defined EC-cell input pathways becomes physiologically meaningful only if these signals are converted into outputs that can reach the nervous system. Bellono et al. [23] showed that activated EC cells release serotonin in a regulated manner. Using a 5-HT_3_ receptor-based biosensor assay, they found that stimuli engaging TRPA1, Olfr558, or catecholamine-sensitive pathways triggered local 5-HT release, which was markedly reduced by blockade of P/Q-type voltage-gated Ca^2+^ channels [23]. Thus, serotonin functions here not simply as an abundant gut mediator, but as a Ca^2+^-dependent epithelial output that translates diverse sensory inputs into neural-relevant signaling [23].

Bellono et al. [23] also placed this output within a spatially organized epithelial–neuronal context. In 5-HT_3_ receptor reporter mice, 5-HT_3_R-positive nerve fibers extended into intestinal villi and closely apposed EC cells. The authors further described synaptic-like epithelial–neuronal associations, suggesting that EC-cell serotonin release may occur within restricted signaling microdomains rather than only through diffuse endocrine mechanisms [23]. These findings should be interpreted carefully: they support spatially organized, synaptic-like EC cell–nerve associations, but do not establish that all EC-cell communication occurs through canonical synapses in every intestinal region or context [23].

Most importantly, EC-cell activation was directly linked to sensory afferent physiology. In ex vivo intestinal preparations, EC-cell agonists activated mucosal afferent fibers in a serotonin receptor-sensitive manner and enhanced their mechanosensory responses [23]. These data provide a strong mechanistic bridge between epithelial chemosensation and primary sensory signaling: EC cells can convert chemically diverse gut cues into serotonin-dependent modulation of afferent pathways. However, this evidence remains mechanistic rather than phenotypic. Bellono et al. did not test behavioral pain responses or establish that EC cells themselves generate visceral pain states in vivo; that causal step was addressed in later studies [23].

Together, these findings establish EC cells as a sensory interface between the intestinal epithelium and neural pathways, linking stimulus detection to regulated 5-HT release and afferent modulation. Yet chemical sensing is only part of the problem. Because the mucosa is continuously exposed to deformation, pressure, and shear, the next question is whether EC cells also transduce mechanical force.

### 4.4. Mechanosensory EC-Cell Signaling: Piezo2 as a Force-to-Serotonin Transducer

A mechanistic framework for EC-cell force sensing was provided by Alcaino et al. [57], who asked whether mechanically evoked serotonin release reflects direct EC-cell mechanosensitivity and which molecular machinery mediates this response. Using complementary epithelial and organoid models, they identified Piezo2 as a key mechanotransduction channel in a defined EC-cell subset [57].

Importantly, mechanosensitivity was not generalized to all EC cells. Piezo2 was detected in a subset of murine enteroendocrine and EC cells and localized near 5-HT-containing vesicles, suggesting a direct link between force detection and transmitter release [57]. Mechanical stimulation induced rapid inward currents and Ca^2+^ responses that were reduced by Piezo-type mechanosensitive ion channel component 2 (Piezo2) knockdown or channel inhibition, demonstrating that at least a subpopulation of EC cells is directly mechanosensitive [57].

The most relevant advance for this review was the demonstration of force-to-serotonin conversion. Mechanical stimulation of responsive EC cells triggered Ca^2+^ elevation followed by 5-HT biosensor activation, consistent with serotonin release. These responses were attenuated by Piezo2 silencing or inhibition, while ondansetron blocked the downstream biosensor response without altering the upstream EC-cell Ca^2+^ signal [57]. Conditional epithelial Piezo2 deletion also reduced mechanically stimulated epithelial secretion, supporting physiological relevance at the tissue level [57].

This work extends EC-cell sensory biology from chemical detection to physical deformation, showing that a Piezo2-positive subset can convert mechanical force into serotonergic epithelial output [57]. However, it does not establish a mechanism of mechanical visceral pain, as nociceptive behavior and distension-evoked pain phenotypes were not examined. Rather, it provides a mechanistic substrate through which mucosal deformation or distension-related strain may influence serotonin-dependent sensory signaling in later pain-relevant contexts.

Together, rodent studies define EC cells as electrically excitable, chemosensory, and mechanosensory epithelial transducers. Their translational relevance, however, requires direct examination of primary human EC cells rather than inference from murine models alone.

### 4.5. Human Enterochromaffin Cells as Multimodal Sensory Integrators: Luminal, Neuronal, and Paracrine Control of Serotonin Release

A major translational advance in EC-cell biology came from Alcaino et al. [58], who developed human duodenal organoid systems in which EC cells were genetically labeled at the *TPH1* locus using CRISPR–Cas9 reporters. This enabled purification and detailed functional analysis of primary human EC cells. Although the study did not test visceral pain directly, it provided one of the clearest available views of human EC-cell receptor architecture and secretory control [58].

Human EC cells expressed receptor programs linked to several regulatory domains: luminal nutrient and microbial sensing, gut hormone and paracrine signaling, and neurohumoral input [26,58,59,60,61,62]. These data support a model in which human EC cells function as multimodal epithelial sensory integrators rather than single-purpose chemoreceptors, combining luminal information with local endocrine, paracrine, and neuronal cues [26,58,59,60,61,62].

Importantly, this interpretation was supported functionally. Human EC cells responded to bacterial metabolites, aromatic amino acids, adrenergic agonists, and selected gut hormones through Ca^2+^, cAMP, or secretory responses [58]. Isovalerate increased action potential firing, while acetate and butyrate elicited responses consistent with the receptor repertoire identified transcriptomically [58]. These findings strengthen the broader sensory model of EC cells, while remaining distinct from direct evidence for human pain mechanisms [58].

A key translational point is that human EC-cell regulatory logic is not simply identical to the murine model. In mice, current mechanistic models emphasize defined receptor pathways such as TRPA1-dependent irritant sensing, *Olfr558*-dependent isovalerate sensing, and Adra2A–TRPC4-dependent catecholaminergic activation [23]. In human organoid-derived EC cells, receptor expression and functional responses indicate a partially distinct regulatory hierarchy, including stronger 5-HT secretory responses to β-adrenergic stimulation and a more prominent role for ADRB1-associated signaling [58]. Thus, murine EC-cell pathways should be viewed as mechanistic frameworks rather than direct one-to-one predictors of human EC-cell signaling. The broader principle of neurohumoral EC-cell regulation appears conserved, but the dominant receptor pathways and secretory control mechanisms may be species- and context-dependent [23,58]. This distinction is particularly important when extrapolating EC-cell sensory biology to human visceral pain and disorders of gut–brain interaction.

Taken together, rodent and human studies establish EC cells as polymodal epithelial sensory transducers capable of converting diverse environmental and physiological inputs into regulated serotonergic output. Human organoid models confirm a similarly complex sensory repertoire while revealing species-specific regulatory differences. These data do not yet show that human EC-cell sensory programs drive visceral pain, but they provide an important mechanistic bridge to the next question: whether epithelial sensory activity can shape nociceptive afferent signaling and visceral pain-related phenotypes in vivo.

## 5. From Epithelial Sensory Transduction to Visceral Nociception

### 5.1. Direct Epithelial Activation Is Sufficient to Recruit Extrinsic Afferent Firing and Visceromotor Output

The studies discussed above show that specialized epithelial cells can detect chemically and mechanically diverse stimuli and generate outputs capable of influencing sensory neural pathways [23,26,57,58,59,60,61,62]. The next question was whether epithelial activation can do more than modulate afferent signaling—whether it is sufficient, by itself, to recruit nociception-relevant neural output and evoke a reflex response linked to visceral sensory processing.

Makadia et al. [63] addressed this using an optogenetic strategy that separated epithelial excitation from conventional mechanical or chemical stimulation. Channelrhodopsin-2 was expressed in villin-positive colonic epithelial cells, and Vil-ChR2 mice were examined in ex vivo colon–pelvic nerve preparations with single-fiber recordings of extrinsic afferents [63]. This approach allowed the authors to test epithelial sufficiency directly, without simultaneously activating multiple tissue compartments.

Photostimulation of the colonic epithelium evoked robust firing in extrinsic pelvic afferents. In homozygous Vil-ChR2 mice, approximately 50.5% of colon afferents responded, often with high-frequency discharge resembling responses to physiological mechanical stimuli such as circumferential stretch [63]. Responsive fibers included mucosal, muscular, muscular–mucosal, and serosal classes, indicating that epithelial activation can recruit a broad range of extrinsic sensory pathways [63]. These findings placed the epithelium upstream of afferent activation rather than as a passive surface monitored by nearby nerve endings [63].

The response was at least partly mediated by purinergic signaling. Combined P2X and P2Y receptor blockade reduced or abolished light-evoked firing in most responsive fibers, including 8 of 11 stretch-sensitive afferents [63]. This supports a biologically mediated epithelial–afferent coupling mechanism involving adenosine triphosphate (ATP) and/or uridine triphosphate (UTP), while also indicating that additional epithelial mediators may contribute. The precise signaling molecules and epithelial cellular sources were not resolved [63].

The in vivo experiments extended this principle to reflex-level output. Epithelial photostimulation elicited visceromotor responses similar in form to those induced by colorectal balloon distension [63]. Their longer latency relative to direct activation of transient receptor potential vanilloid 1 (TRPV1)-positive sensory terminals was consistent with an indirect epithelial-to-neural signaling step [63]. Thus, epithelial excitation was sufficient not only to drive extrinsic afferent firing ex vivo, but also to engage a whole-animal reflex commonly used as a preclinical readout of visceral nociceptive processing [63].

These findings require careful interpretation. The villin-driven ChR2 model does not identify the epithelial population responsible for the effect; the response could arise from enterocytes, goblet cells, enteroendocrine cells, EC cells, neuropod-like populations, or coordinated signaling among several compartments. Makadia et al. [63] therefore provide a decisive but cell-type-unresolved demonstration that the colonic epithelium can initiate nociception-relevant afferent and reflex output. The next step is to determine whether a defined epithelial sensory lineage can drive visceral hypersensitivity with causal precision.

### 5.2. Enterochromaffin Cells as Causal Drivers of Acute and Persistent Visceral Hypersensitivity

Bayrer et al. [64] addressed the key cell-type question left unresolved by earlier work. EC cells had already emerged as plausible regulators of pain-relevant signaling because they are electrically excitable, detect aversive or stress-associated stimuli, and communicate with sensory afferents through serotonergic outputs. Makadia et al. [63] had further shown that epithelial excitation can generate afferent and visceromotor responses. Bayrer et al. [64] therefore asked whether EC cells are themselves necessary and sufficient to shape visceral hypersensitivity in vivo.

The study first examined isovalerate, a bacterially derived metabolite used to probe EC-cell-dependent sensory signaling. In ex vivo gut–nerve preparations from Na_V1.8-ChR2 mice, isovalerate increased the responsiveness of mucosal afferents to optical activation in males [64]. Female fibers showed higher baseline sensitivity and did not display the same additional enhancement. Because Channelrhodopsin expression in DRG neurons did not differ between sexes, the divergence was unlikely to reflect transgene expression alone [64]. These data suggest sex-dependent baseline states of the EC-cell–mucosal afferent axis, potentially with greater tonic engagement in females, although they should not be extrapolated into a definitive model of sex differences in human visceral pain [64,65,66].

Bayrer et al. [64] next showed that intracolonic isovalerate recruits spinal sensory neurons in vivo. Intravital calcium imaging of lumbosacral dorsal root ganglion (DRG) neuronrevealed isovalerate-responsive cells in both sexes, with responses detected in 6.5% of neurons in males and 2.2% in females, while basal DRG activity was higher in females [64]. In males, the 5-HT_3_ receptor antagonist alosetron attenuated isovalerate-evoked DRG activation, supporting a partial serotonergic contribution under these conditions. However, 5-HT_3_ signaling did not fully account for the response or the sex-dependent differences [64].

This neural recruitment was accompanied by a nociception-relevant reflex phenotype. Intracolonic isovalerate enhanced visceromotor responses to colorectal distension in males across several pressures, without altering colonic compliance [64]. The same effect was less evident in females, consistent with their higher baseline sensory activity. These findings indicate that a microbial metabolite capable of engaging EC-cell sensory pathways can induce experimental visceral hypersensitivity. As throughout this review, visceromotor responses should be interpreted as preclinical reflex-level readouts of visceral sensitivity, not as direct equivalents of subjective pain experience [64].

The strongest evidence came from targeted tests of EC-cell necessity and sufficiency. Selective suppression of vesicular release from EC cells using tetanus toxin light chain reduced stimulus-evoked 5-HT release in organoids, lowered baseline visceral sensitivity, and abolished the robust isovalerate-induced enhancement of distension-evoked visceromotor responses observed in control males [64]. Thus, EC-cell signaling was required for key components of the hypersensitivity phenotype.

Conversely, chemogenetic activation of EC cells increased 5-HT release, sensitized mucosal afferent responses to mechanical stimulation, and enhanced visceromotor responses to colorectal distension in males, with a weaker trend in females [64]. In males, alosetron prevented the deschloroclozapine (DCZ)-induced increase in visceromotor responses, again supporting a serotonergic component. These experiments establish that selective EC-cell activation is sufficient to generate visceral hypersensitivity in the tested models [64].

Bayrer et al. [64] also showed that repeated EC-cell activation can produce a persistent sensitized state. After daily DCZ administration for 21 days and a 72 h washout, male EChM3Dq mice retained elevated visceromotor responses to colorectal distension [64]. This indicates that sustained EC-cell activation can generate hypersensitivity that outlasts immediate stimulation. However, whether this “colitis-free” paradigm constitutes a fully developed model of chronic pain remains unresolved. The most careful interpretation is that persistent epithelial sensory perturbation can help maintain a sensitized visceral state beyond the period of direct activation [64].

Taken together, Bayrer et al. [64] provide direct causal evidence that a defined epithelial sensory lineage can drive the emergence and persistence of visceral hypersensitivity in vivo. Their study links a metabolite-responsive EC-cell pathway to afferent sensitization, DRG recruitment, and pain-related reflex output, while establishing both necessity and sufficiency through targeted genetic manipulation. These findings raise the next question: which afferent channels decode epithelial pain signals?

### 5.3. Pathway Specificity in Neuroepithelial Pain Signaling: Vagal, Mucosal, and Spinal Afferent Circuits

By this point, “epithelial–neuronal communication” is too broad a term to be analytically useful. Different epithelial sensory populations engage distinct neural channels depending on the stimulus, tissue context, and functional outcome. Nutrient-driven gut–brain signaling, mucosal afferent sensitization, and DRG-linked nociceptive regulation share a neuroepithelial principle, but they are not anatomically or functionally interchangeable.

The best-defined neuropod-cell pathway is the vagal nutrient-sensing circuit. Kaelberer et al. [22] showed that intestinal neuropod cells form synapses with vagal nodose neurons and transmit luminal sugar signals with synaptic temporal precision. This work established that the intestinal epithelium can communicate rapidly and directly with neural pathways projecting toward the brainstem. However, this circuit was defined in the context of nutrient sensing and appetitive gut–brain signaling, not visceral nociception. Its relevance to pain biology is therefore conceptual rather than direct [22].

Bayrer et al. [64] revealed a distinct configuration in visceral hypersensitivity. Their data identify mucosal afferents as prominent downstream targets of EC-cell-dependent sensitization. This is important because mucosal afferents have often been viewed mainly as low-threshold detectors of local tissue deformation rather than as major contributors to pain states. Bayrer et al. [64] show that EC-cell–mucosal afferent signaling can nevertheless play a substantial role in experimental visceral hypersensitivity. This does not exclude the involvement of other nociceptive afferent classes, but it places mucosal afferent pathways more centrally within neuroepithelial models of visceral pain [64].

A further distinction emerges from the GUCY2C neuropod-cell literature developed in the next section. Barton et al. [10] examined GUCY2C-enriched neuropod-like epithelial cells in a visceral pain framework and assessed their functional interaction with dorsal root ganglion sensory neurons rather than vagal nodose neurons. This reinforces a central point: pain-relevant neuropod-cell signaling should not be reduced to the nutrient-sensing vagal circuit described by Kaelberer et al. [22]. Instead, current evidence supports pathway-specific epithelial modules that engage different afferent routes according to biological context.

A cautious synthesis therefore distinguishes three emerging neuroepithelial configurations. First, nutrient-sensing neuropod circuits engage vagal nodose afferents to support rapid gut–brain communication [21,22]. Second, EC-cell pain-sensitizing circuits act prominently through mucosal afferents and downstream spinal sensory signaling to modulate visceral hypersensitivity [23,64]. Third, GUCY2C-enriched neuropod-like pain-regulatory circuits engage DRG-linked sensory pathways and will be examined in detail in the next section [10]. This is not yet a complete wiring diagram of epithelial contributions to visceral pain, but a pathway-specific framework grounded in the current literature.

This distinction matters because pain-relevant epithelial signaling should not be assumed to reuse the circuitry of appetitive gut–brain communication. Section 5 shows that epithelial activation can initiate nociception-relevant output and that EC cells can causally drive acute and persistent hypersensitivity. The next mechanistic question is whether neuropod-like epithelial cells also participate in a distinct DRG-linked pathway that directly regulates visceral pain. This leads to the GUCY2C paradigm.

## 6. Neuropod Cells as Direct Modulators of Visceral Pain: The GUCY2C Paradigm

### 6.1. Before Neuropod Cells: Linaclotide Reveals a GC-C-Dependent Antinociceptive Axis

The preceding section showed that specialized epithelial sensory cells can shape pain-relevant neural signaling and, in defined contexts, contribute to visceral hypersensitivity [10,21,22]. A complementary question is whether epithelial circuits can also act in the opposite direction, restraining sensory gain and suppressing nociceptive output. The clearest example is the guanylate cyclase C pathway, whose relevance to visceral pain was recognized before neuropod cells were implicated in this process [1,67,68].

Guanylate cyclase C, or GC-C, is an intestinal epithelial receptor encoded by *GUCY2C* and activated by endogenous peptide ligands as well as the synthetic agonist linaclotide [1,67,69,70,71,72]. GC-C activation increases intracellular cyclic guanosine monophosphate (cGMP), a signaling event classically linked to epithelial secretion [70,72,73,74]. However, early preclinical studies indicated that this pathway also modulates visceral sensitivity through mechanisms not fully explained by altered fluid handling [70,71,72]. Eutamene et al. [75] provided the first systematic evidence that linaclotide exerts GC-C-dependent antinociceptive effects in experimental visceral hypersensitivity.

Across rodent models of inflammatory, post-inflammatory, and stress-induced hypersensitivity, linaclotide reduced colorectal pain-related responses [75]. In the trinitrobenzene sulfonic acid (TNBS) mode, oral linaclotide decreased abdominal contractions evoked by colorectal distension without correcting altered colonic tone or elasticity [75]. It also had little effect on basal visceral sensitivity in non-hypersensitive animals under the conditions tested, suggesting that its antinociceptive action is most evident in sensitized states rather than as a general suppression of colorectal sensory signaling [75].

Crucially, this effect required intact GC-C signaling. Linaclotide reversed TNBS-induced hypersensitivity in wild-type mice but not in GC-C-null animals [75]. At this stage, neither the relevant epithelial cell type nor the downstream neural mechanism had been identified. Eutamene et al. [75] did not implicate neuropod cells or explain how epithelial receptor activation reduced afferent nociceptive output. Nevertheless, their study established a key conceptual foundation: GC-C activation functions as a receptor-dependent epithelial analgesic axis in experimental visceral hypersensitivity [75].

This finding created the next mechanistic question: if linaclotide acts through an epithelial receptor, how does that epithelial signal suppress nociceptive activity in visceral afferents and the spinal cord?

### 6.2. Epithelial GC-C Signaling Suppresses Colonic Nociceptor Activity: The Castro Model

Castro et al. [76] addressed the mechanistic gap left by earlier pharmacological studies by asking whether epithelial GC-C activation directly suppresses pain-relevant sensory signaling. This distinction was important because linaclotide-mediated antinociception could reflect either active inhibition of nociceptor activity or indirect effects on motility, luminal contents, or tissue mechanics.

Using recordings from high-threshold splanchnic colonic nociceptors in healthy mice and in mice with chronic visceral hypersensitivity, Castro et al. [76] showed that linaclotide reduced mechanically evoked afferent firing, with a stronger effect in the hypersensitive state. These findings linked epithelial GC-C agonism to direct suppression of a pain-relevant electrophysiological output [76].

Several observations indicated that this effect originates in the mucosa rather than through direct neuronal GC-C signaling. GC-C expression was concentrated in the intestinal mucosa, with little or no detectable expression in dorsal root ganglia or spinal cord. Linaclotide failed to inhibit nociceptor firing in GC-C-deficient mice, and removal of the mucosa markedly weakened its effect on colonic afferents [76]. Together, these data supported a model in which linaclotide acts through epithelial GC-C to regulate nociceptor excitability from the mucosal side of the neuroepithelial interface [76].

Castro et al. [76] further proposed a tissue-level mechanism centered on extracellular cGMP. Linaclotide increased epithelial cGMP production and basolateral release, inhibition of cGMP export attenuated nociceptor suppression, and exogenous non-cell-permeant cGMP reduced colonic nociceptor firing, particularly under hypersensitive conditions [76]. These findings support the model: linaclotide → epithelial GC-C activation → basolateral cGMP release → suppression of colonic nociceptor activity. This provided a biologically plausible route by which a mucosal epithelial receptor could modulate extrinsic sensory neurons without requiring direct neuronal GC-C expression [76].

The study also connected peripheral afferent regulation to spinal nociceptive signaling. Intracolonic linaclotide reduced colorectal distension-evoked phosphorylated extracellular signal-regulated kinase (pERK) activation in the thoracolumbar dorsal horn [76]. Although pERK is not a direct measure of subjective pain, its reduction provided an important link between epithelial GC-C activation, diminished afferent drive, and lower spinal nociceptive recruitment [76].

Castro et al. [76] also added translational weight through a post hoc analysis of phase III constipation-predominant irritable bowel syndrome (IBS-C) clinical data, in which a greater proportion of linaclotide-treated patients achieved at least a 30% reduction in abdominal pain than placebo-treated patients [76]. This finding is clinically concordant with the preclinical model, although it does not prove that the full extracellular cGMP–nociceptor mechanism operates identically in patients [76].

Overall, Castro et al. [76] established three key points: epithelial GC-C signaling can suppress pain-related afferent activity; linaclotide analgesia cannot be explained solely by altered secretion or motility; and a clinically relevant epithelial–neuronal analgesic mechanism links mucosal receptor activation to reduced nociceptive output. However, the responsible epithelial cell population remained unresolved, creating the central cellular question later addressed by Barton et al.

### 6.3. GUCY2C-Enriched Neuropod Cells as Specialized Pain-Regulatory Epithelial Nodes

The central advance of Barton et al. [10] was to revisit the GC-C analgesic axis at cellular resolution. Building on Castro’s model [76], the key question became not whether epithelial GC-C signaling can regulate nociception, but which epithelial cells carry this pain-restraining function.

Barton et al. [10] identified a rare population of GUCY2C^high^ intestinal epithelial cells, representing less than 1% of the epithelial compartment and enriched in the proximal small intestine of both mice and humans. In mouse jejunum and human duodenum, these cells showed intense GUCY2C staining and distinctive basal pseudopod-like projections extending toward the lamina propria [10]. This morphology suggested that GC-C-dependent analgesic signaling may be concentrated within a specialized epithelial population positioned for local communication with subepithelial neural elements [10].

Transcriptomic profiling reinforced this interpretation. GUCY2C^high^ cells were enriched for enteroendocrine and neuroendocrine markers, including *Pyy*, *Nts*, *Gcg*, *Sct*, *Gip*, *Chga*, *Cck*, and *Ghrl*, while showing relative depletion of canonical GUCY2C paracrine ligands and components associated with conventional epithelial fluid and electrolyte secretion [10]. They also displayed enrichment of neuronal and synaptic gene programs, including pre- and postsynaptic molecular signatures identified through SynGO analysis [10]. Together with their basal projections and proximity to neuronal structures, these features placed GUCY2C^high^ cells within the broader neuropod-cell framework [10].

This classification requires precision. Barton’s data support the view that GUCY2C^high^ cells are neuropod-like epithelial cells with molecular and morphological features consistent with neuroepithelial signaling, but they should not be treated as equivalent to all previously described neuropod-cell subtypes. In particular, they should not be conflated with the CCK-labeled nutrient-sensing neuropod populations defined in vagal sugar-sensing circuits. The most accurate interpretation is that Barton et al. [10] identified a rare, GUCY2C-enriched neuropod-like epithelial subpopulation positioned to regulate neural activity relevant to visceral nociception [10].

The presence of analogous GUCY2C-enriched epithelial cells with basal projections in human small intestine adds translational relevance [2,10,77]. It indicates that this cellular phenotype is not restricted to mice and provides an anatomical basis for considering related neuroepithelial mechanisms in human gastrointestinal sensory biology [10]. However, these findings do not show that such cells regulate pain in IBS or other disorders of gut–brain interaction. At present, the human data are best viewed as validation of the cellular phenotype rather than disease-specific functional evidence [10].

By assigning a specialized cellular identity to the GC-C analgesic pathway, Barton et al. [10] substantially refined the earlier mucosal model. The relevant epithelial substrate is no longer viewed simply as generic mucosa, but as a rare, GUCY2C-enriched, neuropod-like neuroepithelial population molecularly and morphologically suited to influence neuronal signaling. The next question was whether this cellular specialization has direct functional consequences for pain-relevant sensory neurons.

### 6.4. Neuropod-Cell GUCY2C Signaling Controls DRG Neuron Excitability

To move beyond cellular description, Barton et al. [10] developed an epithelial–neuronal coculture system to test whether GUCY2C^high^ neuropod-like cells can influence the excitability of dorsal root ganglion neurons, a key sensory population in spinal nociceptive pathways. Although this reductionist model does not capture the full complexity of intestinal pain circuits in vivo, it provides a useful platform for isolating epithelial effects on sensory neuron physiology [10].

In the absence of GUCY2C agonism, DRG neurons cocultured with GUCY2C^high^ neuropod-like cells became hyperexcitable, showing reduced rheobase and repetitive firing during sustained depolarization [10]. These features are consistent with enhanced neuronal excitability and indicate that neuropod-like epithelial cells can actively shape the physiological state of pain-relevant sensory neurons rather than merely lie in close proximity to them [10].

Linaclotide reversed this phenotype. In cocultures with GUCY2C-intact epithelial partners, linaclotide restored DRG excitability toward baseline, increasing rheobase and reducing repetitive firing [10]. This effect was lost when the epithelial cells lacked GUCY2C, showing that linaclotide acts through epithelial GUCY2C signaling rather than directly on DRG neurons. These findings provide functional evidence that the GUCY2C^high^ neuropod-like compartment can regulate pain-relevant afferent physiology in a receptor-dependent manner [10].

These experiments also refined the earlier Castro model [76]. In Barton’s coculture system, extracellular cGMP alone did not reproduce the linaclotide-mediated correction of DRG hyperexcitability [10]. This does not contradict Castro’s tissue-level model, in which epithelial GC-C activation promotes cGMP release and suppresses nociceptor activity [76]. Instead, it suggests that the neuropod-associated analgesic mechanism is not fully explained by a simple diffuse extracellular cGMP signal. A more spatially restricted mediator, an additional epithelial signal, or a composite local interaction may be required [10]. Thus, the downstream mediator linking GUCY2C^high^ neuropod-like epithelial cells to reduced DRG excitability remains unknown.

Barton et al. [10] therefore add cellular specificity and mechanistic complexity to the GC-C analgesic pathway defined by Castro et al. [76]. Their data indicate that a rare GUCY2C^high^ neuropod-like epithelial population regulates DRG excitability, and that linaclotide-dependent normalization of this excitability requires epithelial GUCY2C but cannot be fully mimicked by freely applied extracellular cGMP [10,76]. The next step was therefore to determine whether this pathway also restrains visceral nociceptive output in vivo.

### 6.5. Genetic Evidence That Neuropod-Cell GUCY2C Restrains Visceral Nociception

Barton et al. [10] tested the in vivo relevance of this pathway using both global and cell-restricted loss-of-function models. *Gucy2c*-deficient mice showed a heightened visceral nociceptive phenotype, including increased abdominal withdrawal responses to nonnoxious colorectal distension, stronger visceromotor responses to noxious distension, and greater dorsal horn pERK activation after colorectal stimulation [10]. These findings indicate that loss of GUCY2C exaggerates visceral sensory signaling even without a prior experimentally induced hypersensitivity state [10].

These readouts do not measure subjective pain directly, but they are well-established preclinical indicators of visceral nociceptive sensitivity and spinal nociceptive recruitment [10]. The phenotype therefore supports the conclusion that GUCY2C signaling normally restrains visceral nociceptive output. Consistent with this interpretation, linaclotide reduced nociceptive responses in control animals but failed to rescue the elevated sensitivity of *Gucy2c*-deficient mice, confirming that intact GUCY2C is required for linaclotide-responsive suppression of visceral nociception [10].

Because GUCY2C is expressed more broadly across the intestinal epithelium, global receptor deletion could not identify the relevant cellular source of this phenotype [1,10,67,70,72,77]. Barton et al. [10] addressed this limitation by generating *CCK^cre^ Gucy2c^fl/fl^* mice, selectively deleting GUCY2C from a CCK-lineage, neuropod-cell-enriched epithelial population. Immunofluorescence confirmed loss of GUCY2C from Syn1-positive neuropod-like cells, validating the targeted model [10].

This conditional deletion provided the strongest causal evidence in the study. Selective loss of GUCY2C from CCK-lineage neuropod-like cells increased visceral nociceptive signaling and produced linaclotide-refractory phenotypes [10]. These results identify neuropod-cell GUCY2C as a necessary component of the epithelial pain-regulatory pathway, required both to restrain baseline nociceptive output and to preserve responsiveness to GC-C agonism [10].

Taken together, the GC-C/GUCY2C literature forms a coherent mechanistic progression. Eutamene et al. [75] established that linaclotide reduces visceral hypersensitivity through GC-C-dependent pharmacology. Castro et al. [76] then showed that epithelial GC-C signaling suppresses colonic nociceptor activity and downstream spinal activation. Barton et al. [10] added cellular resolution by identifying a GUCY2C-enriched neuropod-like epithelial population, demonstrating its control over DRG excitability, and showing that selective loss of GUCY2C from this compartment disrupts visceral nociceptive homeostasis in vivo. At present, this represents the most direct and mechanistically developed evidence that neuropod-like epithelial cells can regulate visceral pain-related signaling.

The most defensible interpretation is that neuropod-cell GUCY2C functions not as a pain amplifier, but as a homeostatic brake on sensory-neuron hyperexcitability and nociceptive output. This conclusion should not be extended to imply that GUCY2C is the only neuropod-cell pain pathway or that all linaclotide analgesia in vivo is mediated exclusively through CCK-lineage neuropod-like cells. Nevertheless, the current evidence positions the GUCY2C pathway as the strongest model of a pain-restraining neuroepithelial circuit in the gut [10,76]. Its causal strength also makes the unresolved mechanistic questions especially important.

### 6.6. The GUCY2C Model as a New Neuroepithelial Pain Framework: Unresolved Mechanisms and Translational Questions

Barton et al. [10] provide the strongest current evidence that neuropod cells regulate visceral nociception. Their GUCY2C model not only establishes a pain-restraining epithelial pathway, but also exposes the key mechanistic and translational questions that now define the field: what signal is transmitted, how is the epithelial–neuronal interaction organized, and does this mechanism operate in human disease?

A first unresolved issue is the identity of the downstream mediator linking GUCY2C^high^ neuropod-like epithelial cells to reduced DRG excitability. Barton’s coculture experiments showed that extracellular cGMP alone did not reproduce the corrective effect of linaclotide on DRG hyperexcitability [10]. Thus, the analgesic signal downstream of epithelial GUCY2C activation remains incompletely defined and should not be reduced to freely diffusible extracellular cGMP alone. The relevant output may involve an unidentified secreted mediator, spatially constrained cGMP signaling, a coupled transmitter system, or a more complex local epithelial–neuronal interaction. At present, these possibilities remain unresolved.

A second question concerns the mode of neuropod cell–DRG communication. Earlier nutrient-sensing studies demonstrated rapid synaptic signaling between neuropod cells and vagal neurons [40,78,79], but Barton’s pain-regulatory model does not establish that GUCY2C^high^ neuropod-like cells communicate with DRG neurons through a canonical synapse [10]. The available data support functional epithelial–DRG coupling, yet whether this interaction is synaptic, paracrine, or hybrid remains unknown. This distinction matters because nutrient-sensing vagal circuits and pain-relevant spinal afferent pathways should not be assumed to use the same communication logic [10].

A third unresolved issue is anatomical organization. Barton et al. [10] found that GUCY2C^high^ neuropod-like cells are particularly enriched in the proximal small intestine of mice and humans, whereas several nociceptive readouts relied on colorectal distension. This spatial mismatch remains unexplained. It may reflect less abundant distal cell populations, broader system-level control of visceral afferent tone, or a distributed homeostatic mechanism, but none of these interpretations has yet been demonstrated [10].

Human disease relevance also remains open. Barton et al. [10] identified GUCY2C^high^ neuropod-like epithelial cells in human small-intestinal tissue, and Castro et al. [76] linked linaclotide treatment to improved abdominal pain outcomes in post hoc IBS-C clinical analyses. Together, these findings form an important translational bridge, but they do not yet prove that human neuropod-cell GUCY2C signaling regulates IBS-associated pain. Addressing this gap will require human epithelial–sensory neuron cocultures, patient-derived models, disease-tissue mapping of GUCY2C^high^ neuropod-like cells, and functional links to therapeutic responsiveness.

More broadly, the GUCY2C paradigm supports a bidirectional model of epithelial control over visceral sensation. EC cells can amplify hypersensitivity, whereas GUCY2C-enriched neuropod-like cells appear to restrain nociceptive gain [10]. This does not define a single universal neuroepithelial circuit, but it suggests that specialized epithelial lineages may either escalate, buffer, or stabilize sensory output depending on cell identity, molecular pathway, and physiological context. The GUCY2C paradigm therefore extends neuropod-cell biology from nutrient sensing to direct regulation of visceral nociception [10,30,40,70,72,78,80]. It provides the strongest current evidence that a specialized epithelial population can restrain pain-related afferent hyperexcitability and modulate preclinical nociceptive output [21,22,30,63,81,82]. Clarifying the mediator, communication mode, anatomical organization, and human disease relevance of this analgesic neuroepithelial pathway should now be a central priority for visceral pain biology.

To place these developments in a broader historical and mechanistic context, Table 1 summarizes the primary studies that progressively established the neuroepithelial framework developed in this review. The table traces the field from early evidence for epithelial GC-C-dependent antinociception and enteroendocrine structural specialization, through the discovery of rapid epithelial-to-neural sensory transmission, to recent demonstrations that defined epithelial cell populations can amplify or restrain visceral nociceptive signaling. Rather than serving as a comprehensive bibliography, the table highlights the key experimental advances that collectively shifted the field from epithelial sensory plausibility to direct pain-relevant neuroepithelial mechanisms.

## 7. Integrative Model: Epithelial Circuits as Amplifiers, Filters, and Brakes of Visceral Pain

### 7.1. Two Complementary Epithelial Pain Modules: Amplification Versus Restraint

The evidence assembled above argues against a one-directional model in which epithelial sensory cells are viewed simply as initiators of visceral pain [10,76]. Instead, distinct epithelial lineages appear to influence nociceptive signaling in opposite ways. Enterochromaffin (EC) cells can increase afferent excitability and promote visceral hypersensitivity, whereas GUCY2C-enriched neuropod-like cells can restrain sensory neuron hyperexcitability and limit pain-related output [10,64]. These findings support a modular view of the intestinal epithelium, in which the direction of sensory influence depends on cell identity, molecular output, and circuit context.

**Figure 1:**
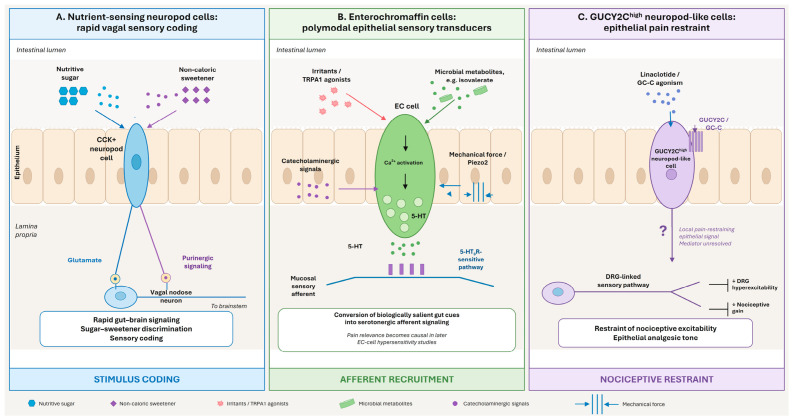
Specialized epithelial sensory circuits at the gut–neural interface. Specialized intestinal epithelial sensory cells engage distinct neural pathways and perform non-equivalent functions in gut sensory biology. (**A**) CCK-labeled nutrient-sensing neuropod cells discriminate nutritive sugars from non-caloric sweeteners and route these inputs through stimulus-dependent transmitter outputs toward vagal nodose pathways, supporting rapid gut–brain signaling and epithelial sensory coding. (**B**) Enterochromaffin cells act as polymodal epithelial sensory transducers, integrating irritant, microbial, catecholaminergic, and mechanical cues into Ca^2+^-dependent serotonin release and 5-HT_3_R-sensitive mucosal afferent signaling. Although these mechanisms establish pain-relevant afferent coupling, causal amplification of visceral hypersensitivity is demonstrated in later EC-cell studies. (**C**) GUCY2C^high^ neuropod-like epithelial cells define a distinct pain-restraining module. Linaclotide/GC-C agonism engages this epithelial pathway, which is associated with reduced DRG-linked hyperexcitability and nociceptive gain through a local inhibitory signal whose precise mediator remains unresolved. Together, these modules support a model in which the intestinal epithelium can contribute to stimulus coding, afferent recruitment, and nociceptive restraint.

These emerging epithelial sensory modules can be organized into three conceptually distinct, though biologically related, neuroepithelial configurations (Figure 1). Nutrient-sensing neuropod cells provide the clearest model of stimulus-selective epithelial coding within rapid vagal gut–brain communication. Enterochromaffin cells illustrate how polymodal epithelial transduction can recruit serotonin-sensitive afferent signaling. GUCY2C^high^ neuropod-like epithelial cells, in turn, define a pain-restraining neuroepithelial pathway linked to reduced nociceptive excitability. Viewed together, these systems support a broader model in which specialized epithelial cells do not perform a single sensory function, but instead contribute differently to the classification, recruitment, or restraint of neural signals arising from the gut.

The clearest pain-amplifying module currently emerges from EC-cell biology. Bellono et al. [23] showed that EC cells are electrically excitable epithelial chemosensors that detect irritants, microbial metabolites, and catecholaminergic signals, convert these inputs into regulated serotonin release, and engage sensory neural pathways [23]. Alcaino et al. [57] extended this framework by demonstrating Piezo2-dependent mechanotransduction in a subset of EC cells, linking epithelial deformation to serotonin release [57]. Together, these studies positioned EC cells as polymodal epithelial transducers capable of detecting several stimulus classes plausibly relevant to nociceptive escalation [23,57,84].

Bayrer et al. [64] then provided strong causal evidence that EC-cell signaling can amplify visceral hypersensitivity. In their models, EC-cell-dependent activity sensitized mucosal afferents, recruited dorsal root ganglion activity in vivo, and enhanced visceromotor responses to colorectal distension. EC-cell silencing reduced key hypersensitivity phenotypes, whereas chemogenetic EC-cell activation was sufficient to induce them. Repeated activation further produced a sensitized state that persisted after stimulation had ceased [64]. EC-cell circuits therefore represent the strongest current example of epithelial pathways that can increase nociceptive gain under defined experimental conditions.

The sex-dependent pattern in this study is important for translational interpretation. Across several assays, females showed higher baseline sensory activity or visceral sensitivity, whereas males displayed a larger dynamic increase after isovalerate exposure or EC-cell activation in the experimental setting [64]. One possible interpretation is that the EC-cell–afferent axis may operate against different baseline gain states in males and females, with a more tonically engaged sensory circuit in females. This observation is relevant because irritable bowel syndrome and chronic abdominal pain disorders are more prevalent in women [6,85,86,87]. However, these preclinical findings should not be treated as a direct explanation for sex differences in human IBS. Rather, they identify sex as a biological variable that may shape epithelial–afferent gain and should be incorporated systematically into future neuroepithelial pain studies [64].

This interpretation is reinforced by broader epithelial sufficiency studies, although without cell-type resolution. Makadia et al. [63] showed that optogenetic activation of the colonic epithelium evokes extrinsic colonic afferent firing and visceromotor responses, with a partially purinergic component. Because the epithelial activation strategy was broad, the responsible cell population remained unresolved [63]. Nevertheless, the study established an important principle: epithelial activation alone can initiate nociception-relevant afferent and reflex output [63].

The complementary module is epithelial restraint, most clearly exemplified by the GUCY2C pathway. Castro et al. [76] showed that linaclotide-dependent activation of epithelial GC-C suppresses colonic nociceptor firing, reduces dorsal horn pERK activation, and operates through an epithelial signaling mechanism involving extracellular cGMP [76]. Barton et al. [10] refined this model by identifying rare GUCY2C^high^ neuropod-like epithelial cells as pain-regulatory nodes. These cells influence dorsal root ganglion neuron excitability, and loss of neuropod-cell GUCY2C increases nociceptive output and produces linaclotide-refractory phenotypes in vivo [10]. The GUCY2C neuropod-like cell paradigm therefore provides the strongest current evidence that a specialized epithelial sensory lineage can function as an endogenous brake on visceral nociception.

Taken together, these findings support a dual-module model of epithelial control over visceral pain. EC-cell pathways currently exemplify epithelial amplification of nociceptive signaling, whereas GUCY2C-enriched neuropod-like pathways exemplify epithelial restraint or gating of nociceptive gain. This framework should not be interpreted as a definitive taxonomy of all epithelial pain-regulatory cells, nor as evidence that every EC cell is pain-promoting or every neuropod-like cell is analgesic. Rather, it is an evidence-based organizing model in which epithelial effects on afferent output depend on cell identity, mediator repertoire, receptor state, afferent pathway, and tissue context [10,64,76]. The key question is therefore no longer whether epithelial sensory cells influence visceral pain, but how distinct epithelial lineages differentially tune nociceptive gain.

Because these epithelial sensory systems are mechanistically related but biologically non-equivalent, it is important to distinguish them at the level of cell identity, sensory input, neural partner, and pain relevance. Table 2 provides a cell-population-centered synthesis of the major epithelial sensory modules discussed in this review. It separates nutrient-sensing CCK-labeled neuropod cells, GUCY2C^high^ neuropod-like pain-regulatory cells, murine and human enterochromaffin cells, and the broad villin-positive epithelial compartment used in epithelial sufficiency experiments. This comparison clarifies that neuroepithelial regulation of visceral sensation does not arise from a single generic epithelial “sensory cell”, but from distinct epithelial populations with different signaling programs and different levels of evidence for involvement in pain biology.

### 7.2. From Signal Detection to Sensory Coding: Epithelial Cells as Classifiers of Biological Meaning

Once epithelial sensory lineages are recognized as capable of either amplifying or restraining nociceptive output, a deeper conceptual question emerges: how do these cells shape which biological signals are transmitted, escalated, or buffered before they reach afferent pathways? The available literature suggests that specialized epithelial sensory cells are not simple binary detectors. Rather, they can preserve stimulus identity, route related inputs through distinct output programs, and thereby influence the neural meaning of intestinal information. This broader principle may be captured by the concept of epithelial sensory coding.

The neuropod-cell literature provides the clearest precedent. Kaelberer et al. [22] showed that neuropod cells rapidly transmit glucose-related luminal information to vagal circuits, establishing a direct epithelial route for structured gut-to-brain signaling [22]. Buchanan et al. [41] then demonstrated that duodenal neuropod cells distinguish nutritive sugars from non-caloric sweeteners and route these inputs through different transmitter outputs: glutamatergic signaling for sugar-related information and purinergic signaling for sweetener-related information [41]. These findings show that epithelial sensory cells can encode qualitative differences between closely related luminal cues rather than functioning as nonspecific nutrient sensors.

A related principle emerges from EC-cell biology. Bellono et al. [23] showed that EC cells detect biologically distinct chemical signals, including irritants, microbial metabolites, and catecholaminergic cues, and convert them into regulated serotonergic outputs directed toward sensory pathways [23]. Alcaino et al. [57] extended this repertoire by demonstrating Piezo2-dependent mechanosensory serotonin release [57]. Together, these findings position EC cells as polymodal epithelial integrators of chemical, microbial, neurohumoral, and mechanical information. Although these inputs converge on EC-cell output, they are biologically distinct, raising the possibility that EC cells contribute to classifying different categories of intestinal signals.

Pain-focused studies show that epithelial signal processing can carry different consequences for nociceptive output. Bayrer et al. [64] demonstrated that a microbial metabolite-associated EC-cell pathway can progress to afferent sensitization, DRG recruitment, and persistent visceral hypersensitivity [64]. Barton et al. [10] revealed the complementary possibility: GUCY2C signaling in neuropod-like epithelial cells shapes DRG excitability and nociceptive tone, with intact signaling restraining hyperexcitability [10]. Although Barton et al. [10] did not address sensory coding in the stimulus-discrimination sense established by Buchanan et al. [41], their findings show that the molecular state of a specialized epithelial population can influence whether downstream afferent pathways remain constrained or become pathologically hyperexcitable.

Epithelial sensory coding can therefore be understood as the capacity of specialized gut epithelial sensory cells to preserve biologically meaningful distinctions among luminal, microbial, mechanical, or homeostatic signals and route them into specific neural or neurohumoral outputs. The clearest evidence for stimulus-specific epithelial coding comes from Kaelberer et al. [22] and Buchanan et al. [41]; the strongest evidence for polymodal sensory integration comes from Bellono et al. [23] and Alcaino et al. [57]; and the clearest evidence that epithelial signaling can shape pain-related outcomes comes from Bayrer et al. [64] and Barton et al. [10].

This concept should be treated as an integrative framework rather than a fully mapped epithelial “code” for visceral pain. Epithelial sensory processing does not determine conscious pain perception in isolation from spinal, supraspinal, immune, vascular, and tissue-state mechanisms. Nevertheless, the existing literature supports the idea that epithelial sensory cells contribute to early biological signal classification and afferent routing. In this sense, the epithelial layer may function not simply as a sensory surface, but as a regulatory interface capable of filtering, amplifying, or restraining neural output before it enters pain-relevant pathways.

### 7.3. Toward a Functional Model of Epithelial Control over Visceral Nociception

Taken together, the literature supports a revised view of the gut epithelium. Beyond serving as a physical barrier and endocrine interface, it can be understood as a signal-processing layer positioned upstream of visceral afferent gain control. This perspective does not replace established neural, immune, or stromal models of gut sensation. Rather, it adds an earlier regulatory tier at the mucosal boundary, where specialized epithelial cells detect, classify, and transform biologically meaningful stimuli before they are integrated into peripheral and central sensory circuits.

Within this framework, epithelial sensory circuits can be organized functionally as amplifiers, filters, and brakes. Amplifiers are exemplified most clearly by EC-cell circuits, in which chemically or mechanically salient cues are converted into serotonin-dependent afferent recruitment and, under defined experimental conditions, visceral hypersensitivity [23,57,64]. Filters or classifiers are illustrated by neuropod-cell discrimination of nutritive sugars from non-caloric sweeteners and by the broader capacity of EC cells to distinguish among irritant, microbial, catecholaminergic, and mechanical inputs [22,23,41,58]. Brakes are represented most strongly by GUCY2C-enriched neuropod-cell signaling, which limits DRG hyperexcitability and preserves linaclotide-responsive suppression of nociceptive output [10,76].

Importantly, these functional categories differ in evidentiary strength. EC-cell amplification is directly supported by causal experiments in visceral hypersensitivity models, and GUCY2C-enriched neuropod-like signaling is causally supported as a pain-restraining pathway in mice. By contrast, neuropod-cell filtering or classification is currently best supported as a mechanistic precedent from nutrient-sensing studies; whether analogous epithelial filtering operates for aversive or nociceptive gut signals remains to be determined.

This three-part architecture is not a single experimentally demonstrated circuit. It is a functional model that organizes currently fragmented mechanistic findings into a coherent conceptual framework. Its value lies in moving beyond a simple “epithelium as pain trigger” narrative and asking how epithelial sensory pathways balance signal detection, afferent escalation, and sensory restraint. From this perspective, visceral hypersensitivity could arise not only from excessive epithelial amplification, but also from impaired epithelial braking, distorted sensory classification, or maladaptive coupling between epithelial outputs and afferent pathways. These possibilities remain to be tested directly, but they emerge logically from the evidence reviewed here.

Neuroepithelial pain biology is therefore best understood not as a search for a single epithelial “pain sensor”, but as the study of a distributed epithelial regulatory layer that can classify intestinal signals, amplify nociceptive salience, and restrain aberrant sensory gain.

## 8. Translational Implications: Epithelial Sensory Circuits in Chronic Visceral Pain Disorders

### 8.1. Disorders of Gut–Brain Interaction as the Principal Clinical Framework for Neuroepithelial Pain Biology

The conceptual model developed above—that epithelial sensory circuits can amplify, filter, or restrain visceral nociceptive signaling—becomes particularly relevant in chronic abdominal pain disorders [10,57,64]. Disorders of gut–brain interaction, especially irritable bowel syndrome, provide the clearest current translational setting. Abdominal pain is central to IBS, yet its severity and persistence are not always explained by overt structural pathology [6,84,85,86,87,88]. This creates a mechanistic space in which altered epithelial–afferent communication may matter: luminal chemistry, microbial metabolites, epithelial receptor state, and transmitter release could all modify visceral sensory gain without frank tissue destruction [89,90,91,92,93].

Clinical observations support the relevance of this framework, although they do not yet define a complete epithelial-cell mechanism in patients. Visceral hypersensitivity is associated with gastrointestinal symptom severity in functional gastrointestinal disorders, including IBS, and psychological distress and altered transit can further shape patient-reported outcomes [85,87]. In IBS-C, GC-C agonist therapy provides the clearest clinically anchored link between epithelial receptor signaling and abdominal pain improvement. Castro et al. [76] reported, in a post hoc analysis of phase III IBS-C trial data, that a greater proportion of linaclotide-treated patients achieved clinically meaningful abdominal pain reduction compared with placebo-treated patients. Related GC-C agonist therapy has also been associated with improvement of abdominal and bowel symptoms in IBS-C [94]. These data support the therapeutic relevance of epithelial receptor pathways in human abdominal pain, but they do not prove that GUCY2C^high^ neuropod-like cells or EC-cell sensory programs directly drive IBS pain. The key translational gap is therefore to connect epithelial sensory cell states in patient-derived tissue or organoids with visceral sensitivity, symptom severity, microbial or metabolite profiles, and treatment responsiveness.

Epithelial sensory dysfunction should not be viewed as a replacement for immune, enteric, spinal, or supraspinal mechanisms of chronic visceral pain. Rather, it offers an upstream regulatory layer that may help explain how subtle mucosal changes produce disproportionately strong sensory consequences. Preclinical studies support this possibility [2,68,89,90,91,92]. Bayrer et al. [64] showed that EC-cell activation sensitizes mucosal afferents, recruits DRG activity, enhances visceromotor responses to colorectal distension, and can generate persistent hypersensitivity after repeated activation. In the opposite direction, Barton et al. [10] demonstrated that loss of epithelial GUCY2C signaling, including within a neuropod-cell-enriched lineage, increases visceral nociceptive output and produces linaclotide-refractory phenotypes. Together, these findings suggest that chronic abdominal pain may, in some contexts, reflect an altered balance between epithelial pathways that amplify afferent excitability and those that normally restrain it.

The strongest current translational bridge is provided by the GC-C/GUCY2C–linaclotide axis. Castro et al. [76] showed that linaclotide acts through epithelial GC-C to suppress mechanically evoked firing of colonic nociceptors and reduce colorectal distension–evoked dorsal horn pERK activation, linking epithelial receptor signaling to attenuation of both peripheral and spinal nociceptive output [76]. These findings do not establish the complete GC-C–cGMP–nociceptor mechanism in human IBS-C, but they provide clinically concordant support for the idea that epithelial receptor signaling can influence abdominal pain, not only secretion or bowel habit [1,72,93,94].

Barton et al. [10] further refine this translational framework by identifying a rare GUCY2C-enriched neuropod-like epithelial population capable of regulating DRG excitability. Linaclotide normalized neuropod-associated neuronal hyperexcitability only when epithelial GUCY2C was intact [10]. This raises an important hypothesis: responsiveness to epithelial analgesic agents may depend, at least in part, on the molecular state of specialized epithelial sensory cells rather than on bulk mucosal receptor expression alone. This remains untested in human disease, but it shifts the translational question from whether epithelial signaling can affect pain to whether specific epithelial circuit states shape pain vulnerability and treatment response.

Disorders of gut–brain interaction therefore provide both a clinically relevant setting and a conceptual test case for the framework developed here. The central translational hypothesis is not that IBS arises from a single epithelial defect, but that chronic visceral pain may, in selected contexts, emerge partly from maladaptive epithelial regulation of afferent gain. This leads directly to the next question: which upstream factors tune the excitability, mediator capacity, or sensory bias of these epithelial circuits?

### 8.2. Microbial Metabolites as Modulators of Epithelial Sensory Tone and Pain-Relevant Signaling

If epithelial sensory circuits shape visceral afferent gain, then the luminal chemical environment and microbial metabolites become important upstream regulators. These factors can continuously and locally influence epithelial cell state at the earliest interface between the gut lumen and the nervous system [95,96,97,98,99]. Their relevance, however, should be framed cautiously. Current evidence does not support a simple claim that microbial metabolites directly cause visceral pain through EC cells [1,68,72,100]. A more defensible interpretation is that microbial ecology can shape epithelial sensory tone and thereby influence how readily pain-relevant neuroepithelial signaling is engaged.

Yano et al. [83] provided the foundational evidence for this concept. Germ-free mice showed markedly reduced colonic, fecal, and circulating serotonin, accompanied by lower colonic *Tph1* expression, indicating impaired EC-cell serotonergic capacity rather than loss of enteroendocrine cells. Recolonization with spore-forming bacterial communities restored serotonergic output, while selected fecal metabolites increased serotonin production in chromaffin cell cultures and elevated colonic and circulating serotonin in germ-free animals [83]. These changes also affected gastrointestinal motility and platelet function. Although Yano et al. [83] did not study pain, they established that the microbiota can durably regulate the serotonergic tone of colonic EC cells.

This principle becomes relevant to visceral pain because serotonin is a major EC-cell output capable of engaging sensory pathways. Bellono et al. [23] showed that EC cells respond to biologically salient cues, including electrophilic irritants, catecholaminergic signals, and the microbial metabolite isovalerate, which activates murine EC cells through *Olfr558* and triggers regulated serotonin release linked to sensory neural signaling. Bayrer et al. [64] then showed that a microbial metabolite-associated EC-cell pathway can become directly nociception-relevant: isovalerate sensitized mucosal afferent circuits, recruited DRG activity in vivo, enhanced visceromotor responses to colorectal distension, and participated in an EC-cell-dependent mechanism capable of driving visceral hypersensitivity in experimental models [64].

Together, these studies support a layered model rather than a single established microbiota–pain causal chain. Microbial communities can set baseline EC-cell serotonergic competence, specific microbial metabolites can activate EC-cell sensory pathways, and under defined experimental conditions this signaling can escalate into afferent sensitization and visceral hypersensitivity [23,64,83]. What remains unresolved is whether these levels of evidence converge in human chronic visceral pain disorders as an integrated microbiota–EC-cell–pain axis.

This distinction is important. The microbiota need not be presented as a direct generator of visceral pain to be translationally meaningful. By shaping EC-cell serotonergic tone, microbial communities may alter the epithelial sensory substrate on which pain-relevant signaling later operates. Microbial metabolites could therefore influence both acute EC-cell activation and the baseline readiness of the epithelial sensory layer to amplify, dampen, or reinterpret luminal inputs. This framework generates testable questions: whether chronic visceral pain is associated with altered EC-cell serotonergic tone, whether metabolite signatures correlate with epithelial sensory responsiveness, and whether specific patient subgroups exhibit a higher-gain mucosal sensory state.

These questions define an important translational frontier. Future studies may determine whether microbiota-dependent epithelial sensory tone can identify patient subgroups, yield biomarkers of metabolite-responsive epithelial states, or reveal upstream intervention points before signals are amplified in afferent pathways. The immediate conclusion is not that microbiome manipulation is already a validated epithelial analgesic strategy, but that microbial regulation of epithelial sensory biology deserves a central place in mechanistic models of chronic visceral pain. This naturally raises the next question: whether epithelial sensory circuits can also be targeted therapeutically more directly.

### 8.3. Therapeutic Tractability of Epithelial Pain Circuits: Established Proof of Principle and Emerging Targets

The translational maturity of neuroepithelial pain biology remains uneven across pathways. Some mechanisms already provide clinically anchored proof of principle, whereas others remain compelling preclinical candidates that require substantial validation before they can be viewed as therapeutic strategies [21,23,83,101]. A useful framework distinguishes three tiers: the GC-C/GUCY2C–linaclotide axis as the strongest clinically linked epithelial analgesic pathway; EC-cell serotonergic signaling as a robust preclinical pain mechanism with therapeutic implications; and upstream sensory transducers such as TRPA1, Piezo2, and Olfr558 as mechanistically attractive but clinically immature targets.

The GC-C/GUCY2C–linaclotide pathway is currently the most advanced. Eutamene et al. [75] first showed that linaclotide exerts GC-C-dependent antinociceptive effects in rodent models of visceral hypersensitivity. Castro et al. [76] then linked this pharmacology to an epithelial–neuronal analgesic mechanism: mucosal GC-C activation reduced colonic nociceptor firing, attenuated spinal pERK activation after noxious distension, and aligned with improved abdominal pain outcomes in IBS-C clinical trial data. Barton et al. [10] added cellular resolution by showing that GUCY2C-enriched neuropod-like epithelial cells regulate DRG excitability and that linaclotide normalizes neuropod-associated neuronal hyperexcitability only when epithelial GUCY2C is intact. In vivo, global or neuropod-lineage-restricted loss of GUCY2C increased nociceptive signaling and produced linaclotide-refractory phenotypes [10].

This pathway is translationally distinctive because it connects pharmacology, epithelial mechanism, afferent modulation, in vivo nociceptive readouts, and clinically concordant abdominal pain improvement. It therefore provides the clearest current evidence that epithelial pain-regulatory circuits can be therapeutically tractable. However, its clinical interpretation should remain measured. Barton et al. [10] do not establish that neuropod-cell GUCY2C dysfunction causes human IBS pain, and Castro’s [76] clinical analysis does not prove that the full cGMP–nociceptor mechanism operates identically in patients. The strongest conclusion is that the GC-C/GUCY2C axis offers a highly credible translational paradigm in which epithelial receptor signaling restrains pain-related neural output, and that specialized epithelial cell identity may influence analgesic responsiveness.

A second promising direction involves EC-cell serotonergic coupling to sensory afferents. Bellono et al. [23] showed that EC-cell activation drives serotonin-dependent modulation of sensory pathways, while Bayrer et al. [64] demonstrated that EC-cell signaling can promote nociceptive sensitization and that 5-HT_3_ receptor blockade attenuates specific metabolite-associated neural and behavioral responses in experimental settings [23,64]. These findings identify downstream serotonergic afferent signaling as a plausible route for limiting excessive epithelial amplification of nociceptive gain. Yet this should not be framed as an already validated epithelial-circuit-specific analgesic strategy. Gut serotonin signaling is biologically broad, and current evidence supports mechanistic relevance rather than a direct clinical therapeutic blueprint.

Upstream epithelial sensory transducers are also attractive because they operate before afferent escalation. TRPA1-mediated irritant sensing in EC cells may convert adverse luminal chemistry into epithelial alarm outputs [23]. Piezo2-dependent EC-cell mechanotransduction provides a plausible link between epithelial force sensing and distension-related pain states [57]. *Olfr558*-dependent sensing of isovalerate in murine EC cells connects microbial metabolite recognition to serotonin release and sensory pathway engagement [23]. These pathways are compelling as early transduction nodes, but none should yet be presented as a clinically validated analgesic target in chronic visceral pain disorders. Their translational value remains primarily mechanistic and hypothesis-generating [12,102,103,104].

The next phase of the field requires direct human validation. Patient-derived mucosal tissues, organoid–sensory neuron cocultures, and spatially resolved analyses of epithelial sensory cell states will be essential for determining whether experimentally defined circuits are altered in chronic visceral pain disorders. It will also be important to test whether epithelial sensory signatures correlate with pain severity, visceral sensitivity, microbial metabolite profiles, or therapeutic response to linaclotide and related interventions. Species-specific differences in receptor regulation deserve particular attention when murine chemosensory pathways are advanced as translational candidates.

The translational promise of epithelial sensory circuit biology is already tangible through the GUCY2C–linaclotide pathway. The broader challenge is now to determine whether epithelial circuit states can become biomarkers, therapeutic targets, or mechanistic classifiers of chronic visceral pain disorders. More broadly, this field reframes chronic visceral pain not only as a disorder of downstream neural amplification, but also as a potential disorder of upstream epithelial sensory regulation.

## 9. Outstanding Questions and Experimental Priorities

Neuroepithelial pain biology has moved beyond the foundational question of whether intestinal epithelial sensory cells can engage neural pathways. That principle is now supported across several experimental systems, including neuropod-cell communication with vagal neurons, EC-cell recruitment of pain-relevant afferent output, and GUCY2C-dependent regulation of nociceptive excitability. The next challenge is to define how these epithelial–neuronal interactions are transmitted, which epithelial populations are involved, which afferent channels they recruit, where they operate along the gut, and whether these mechanisms shape chronic pain states in humans. Resolving these issues will be essential for integrating neuroepithelial circuits into a mature biological framework of visceral pain.

To clarify the current strength of this evidence, Table 3 organizes the field as a hierarchy of evidentiary maturity rather than as a simple chronology of discoveries. It distinguishes structural plausibility, functional epithelial-to-neural communication, epithelial sufficiency for nociception-relevant output, causal lineage-specific control of visceral hypersensitivity, and clinically anchored analgesic relevance. This framework helps define where the neuroepithelial pain model is already experimentally robust and where important mechanistic or translational gaps remain.

### 9.1. Synaptic, Paracrine, or Hybrid Communication? Defining the Mode of Epithelial–Neuronal Signaling

The existence of epithelial–neuronal communication is no longer in doubt; the unresolved issue is its mode. Bohórquez et al. [21] identified sensory enteroendocrine cells with basal projections, synaptic-associated molecular machinery, and traceable epithelial–neuronal connectivity. Kaelberer et al. [22] then provided the strongest functional benchmark by showing that neuropod cells transmit sugar-related signals to vagal nodose neurons with millisecond-scale kinetics through glutamatergic signaling. Together, these studies establish that epithelial sensory transduction can, in defined nutrient-sensing circuits, operate with synaptic temporal precision [21,22].

This neuropod–vagal paradigm should not, however, be treated as a universal model for all epithelial sensory pathways. EC cells release serotonin in response to defined chemical cues and couple to 5-HT_3_R-sensitive sensory pathways, with evidence for spatially organized EC-cell–nerve associations [23]. Yet whether EC-cell afferent signaling is primarily synaptic, short-range paracrine, or context-dependent remains unresolved. This distinction matters because the geometry and duration of mediator exposure may strongly influence afferent gain in pain-relevant settings [23].

The GUCY2C pathway introduces a further mechanistic gap. Barton et al. [10] showed that GUCY2C^high^ neuropod-like epithelial cells regulate DRG neuron excitability and that linaclotide normalizes this phenotype in a GUCY2C-dependent manner. However, extracellular cGMP alone did not reproduce the corrective effect observed in coculture, despite the earlier Castro model linking epithelial GC-C activation to extracellular cGMP-mediated nociceptor inhibition [10,76]. This suggests that the neuropod-like analgesic mechanism is more organized than a simple freely diffusible mediator acting uniformly in the extracellular space. Importantly, Barton et al. do not establish a canonical neuropod–DRG synapse; they expose a mechanistic question that now requires direct resolution.

A key priority is therefore to determine whether epithelial pain circuits use synaptic, paracrine, juxtacrine, or hybrid signaling modes, and whether these vary by epithelial cell type, stimulus class, tissue region, and afferent target. This issue will shape how neuroepithelial circuits are mapped, how coculture systems are interpreted, and how epithelial pain pathways are ultimately targeted therapeutically.

### 9.2. Resolving Pathway Specificity: Vagal, Mucosal, and DRG-Linked Neuroepithelial Circuits

Neuroepithelial circuit identity depends not only on the epithelial cell involved, but also on the afferent pathway engaged. A neuropod cell transmitting nutrient information to vagal nodose neurons, an EC cell sensitizing mucosal afferents, and a GUCY2C^high^ neuropod-like cell modulating DRG excitability should not be treated as interchangeable versions of one epithelial sensory system.

The best-defined epithelial–afferent circuit remains the vagal nutrient-sensing pathway. Kaelberer et al. [22] demonstrated rapid transmission from intestinal neuropod cells to vagal nodose neurons, and Buchanan et al. [41] showed that duodenal neuropod cells distinguish nutritive sugars from non-caloric sweeteners through stimulus-specific transmitter outputs [41]. These studies define a precise vagal information channel, but they do not establish a visceral nociceptive pathway.

Pain-focused studies point instead toward mucosal and DRG-linked signaling. Bayrer et al. [64] showed that EC-cell activity can sensitize mucosal afferents, recruit DRG activity in vivo, enhance visceromotor responses, and generate persistent hypersensitivity after prolonged activation. Barton et al. [10] identified a distinct pain-restraining epithelial route in which GUCY2C^high^ neuropod-like cells regulate DRG excitability, while loss of GUCY2C enhances nociceptive output and weakens linaclotide responsiveness [10]. These findings suggest that the strongest current epithelial pain models are more closely aligned with mucosal afferent and DRG-associated pathways than with the vagal circuits established in nutrient sensing.

Makadia et al. [63] further showed that broad optogenetic activation of villin-positive colonic epithelium is sufficient to recruit extrinsic afferent firing and visceromotor responses. However, the epithelial subtype responsible was not resolved [63]. This study therefore demonstrates epithelial sufficiency while also emphasizing the need for cell-type-specific circuit mapping.

The next major step is to build a regionally and cell-type-resolved neuroepithelial wiring map. Such a framework should connect defined epithelial populations with identified afferent subclasses, distinguish vagal from spinal sensory routes, and account for regional differences between small intestine, proximal colon, and distal colon.

### 9.3. Defining Epithelial Sensory Cell Identity: What Qualifies as a Neuropod Cell?

The conceptual reach of neuropod-cell biology now exceeds the precision of its taxonomy. The field no longer needs to establish that neuropod-like epithelial cells exist; it must determine how broadly or narrowly this category should be defined.

Several related but non-identical epithelial populations now occupy this conceptual space. Bohórquez et al. [21] described sensory enteroendocrine cells with basal projections, synaptic-associated machinery, and direct neural connectivity. Kaelberer et al. [22] functionally defined neuropod cells as rapid epithelial sensory transducers within a vagal nutrient-sensing circuit. Buchanan et al. [41] focused on CCK-labeled duodenal neuropod cells capable of sugar–sweetener discrimination through transmitter-selective outputs. Barton et al. [10] then identified rare GUCY2C^high^ neuropod-like epithelial cells with neuroendocrine and synaptic-associated features that regulate DRG excitability and visceral nociceptive output [10].

These populations clearly belong to a shared neuroepithelial framework, but it remains unresolved whether they represent one broad cell class, overlapping functional states, or several distinct epithelial lineages. In particular, it would be premature to conclude that all neuropod cells are GUCY2C^high^, or that all GUCY2C^high^ cells are equivalent to the CCK-labeled nutrient-sensing neuropod populations described in earlier work [10]. This distinction has direct consequences for genetic targeting, interpretation of cell-specific manipulations, and comparisons between nutrient-related and pain-related epithelial circuits.

EC cells further reinforce the need for clear category boundaries. They are electrically excitable, release vesicular transmitters, couple to sensory pathways, and are directly implicated in visceral hypersensitivity [23,64]. Yet they should not be absorbed into the neuropod-cell category. EC cells and neuropod cells are best viewed as related but biologically distinct epithelial sensory populations with different molecular programs, transmitter repertoires, and pain-relevant functions.

A mature taxonomy of epithelial sensory cells will likely require a composite framework integrating lineage, morphology, regional distribution, transcriptomic state, receptor logic, transmitter repertoire, afferent target, and physiological output.

### 9.4. Human Validation and Disease-Relevant Neuroepithelial Models

Human validation of neuroepithelial sensory biology is advancing, but it remains far less developed than the preclinical literature. Current findings provide important translational footholds. Barton et al. [10] identified GUCY2C^high^ epithelial cells with basal pseudopod-like morphology in human small intestine, supporting the plausibility of a specialized GUCY2C-enriched epithelial population in humans. However, this does not demonstrate that these cells regulate pain, alter human sensory neuron excitability, or contribute directly to IBS or other chronic visceral pain disorders [10].

Human EC-cell biology has progressed further experimentally. Alcaino et al. [58] developed CRISPR-labeled human duodenal organoid-derived EC-cell models and showed that these cells respond to bacterial metabolites, aromatic amino acids, and adrenergic agonists, with isovalerate increasing action potential firing [58]. This work provides a strong platform for human epithelial sensory biology and underscores that receptor logic may not be fully conserved from mouse systems. At the same time, it remains an epithelial sensory model rather than a complete human epithelial–neuronal pain circuit.

The central missing experiment is therefore clear: the field still lacks robust systems that directly test whether human EC cells or human neuropod-like epithelial cells alter human sensory neuron excitability under pain-relevant conditions. Human organoid–sensory neuron cocultures, epithelial–DRG or iPSC-derived nociceptor platforms, patient-derived epithelial models, spatial mapping in disease tissue, and correlations with pain phenotypes or treatment responsiveness should now become priorities.

Equally important, chronic visceral pain cannot be fully understood through acute stimulus–response assays alone. Organoids, cocultures, ex vivo nerve recordings, optogenetics, chemogenetics, in vivo DRG imaging, and visceromotor readouts have been indispensable for mechanism discovery. Yet chronic pain involves prolonged sensory remodeling, persistent epithelial state changes, microbial adaptation, region-specific tissue biology, and interactions among epithelial, neuronal, immune, and stromal compartments. Bayrer et al. [64] showed that repeated EC-cell activation can generate persistent hypersensitivity, Barton et al. [10] identified a GUCY2C-dependent epithelial restraint mechanism whose chronic failure remains an important question, and Yano et al. [84] demonstrated that microbial ecology can durably shape EC-cell serotonergic tone [84]. Together, these findings argue that future models must examine not only acute transduction, but also persistence, maladaptive remodeling, and the longer trajectory from epithelial sensory perturbation to chronic pain.

Disease-relevant platforms should therefore be designed to address specific biological gaps: chronicity, epithelial state persistence, repeated stimulation, regional specificity, microbial context, sex as a biological variable, and multicompartment tissue interactions. The field now needs models capable of asking not only whether an epithelial cell can activate a neuron, but whether an epithelial pathway alters the progression from intestinal stimulus to persistent visceral hypersensitivity.

The next phase of neuroepithelial pain biology should therefore move beyond cataloguing epithelial sensory interactions and toward defining their function within a complete visceral pain logic. The critical question is under which biological conditions epithelial circuits initiate nociception-relevant signaling, amplify afferent gain, filter the biological meaning of intestinal inputs, or restrain aberrant nociceptive escalation. Answering that question will determine whether neuroepithelial circuits become a descriptive addition to gut sensory biology or a mechanistically decisive framework for understanding and treating chronic visceral pain.

## 10. Conclusions

The intestinal epithelium is emerging as more than a physical barrier, endocrine surface, or passive interface surveyed by adjacent nerves. The evidence reviewed here supports a broader view in which specialized epithelial sensory cells form an active neuroepithelial regulatory layer positioned upstream of visceral afferent signaling. Within this layer, epithelial cells do not simply release diffusible mediators into the mucosa; they can detect biologically meaningful stimuli, engage neural pathways with cell-type-specific logic, and influence how intestinal information is transmitted into sensory circuits.

Three lines of work have been especially transformative. Neuropod-cell studies established that epithelial sensory cells can communicate rapidly with vagal neurons and preserve stimulus identity through transmitter-selective coding, providing a foundational model of structured epithelial-to-neural information transfer. Enterochromaffin-cell studies extended this principle into polymodal sensory transduction, showing that chemically, microbially, and mechanically relevant gut cues can be converted into serotonergic afferent signaling and, in experimental models, causally amplify visceral hypersensitivity. The GUCY2C^high^ neuropod-like cell paradigm revealed the complementary possibility that epithelial sensory circuits can also restrain nociceptive gain, regulate DRG excitability, and preserve linaclotide-responsive suppression of pain-related output. Together, these findings support a functional model in which epithelial sensory circuits may act as filters of biological meaning, amplifiers of afferent gain, or brakes on aberrant nociceptive escalation.

This framework does not replace established neural, immune, stromal, spinal, or supraspinal mechanisms of visceral pain. Rather, it adds an upstream epithelial sensory tier that may help explain how luminal, microbial, mechanical, and homeostatic signals are selected, shaped, or constrained before entering pain-relevant pathways. At present, this model is supported mainly by preclinical and experimental evidence and should not be interpreted as proof that epithelial sensory dysfunction explains chronic abdominal pain in humans. Its translational value lies in identifying epithelial circuit states that may contribute to pain vulnerability, persistence, or treatment responsiveness in selected contexts. Defining the cellular logic, molecular mediators, pathway specificity, and human disease relevance of these neuroepithelial circuits will be essential for transforming this emerging field into a mature framework for understanding—and ultimately treating—chronic visceral pain.

## Figures and Tables

**Table 1:** Landmark studies establishing the neuroepithelial framework for gut sensory signaling and visceral pain regulation.

Study	Year	Model/System	Epithelial Population	Neural Partner/Pathway	Key Mechanistic Advance	Direct Pain Relevance
Eutamene et al. [75]	2010	Rodent visceral hypersensitivity models; GC-C-null mice	GC-C-expressing intestinal epithelium	Not directly resolved	Linaclotide produces GC-C-dependent antinociception	Direct experimental visceral hypersensitivity
Castro et al. [76]	2013	Mouse nociceptor recordings; dorsal horn pERK; epithelial cGMP assays; IBS-C post hoc analysis	GC-C-expressing mucosa	High-threshold colonic nociceptors; spinal nociceptive signaling	GC-C → extracellular cGMP → nociceptor inhibition	Clinically concordant analgesic relevance
Bohórquez et al. [35]	2014	Mouse intestine; 3D electron microscopy; organoids	Neuropod-bearing EECs	Enteric glial association; neuronal pathway not directly demonstrated	Neuropods identified as vesicle-rich basal projections	None—foundational sensory biology
Bohórquez et al. [21]	2015	Reporter mice; nerve mapping; coculture; rabies tracing	Sensory EECs with neuropods	Mucosal nerve fibers/traceable epithelial-to-neural circuit	Neuroepithelial circuit concept established	None—foundational sensory biology
Yano et al. [83]	2015	Germ-free mice; microbial recolonization; metabolite exposure	Colonic EC cells	Not directly tested; microbiota-dependent EC-cell serotonergic tone	Microbiota regulate EC-cell 5-HT biosynthesis	Indirect/conceptual
Bellono et al. [23]	2017	Murine organoids; electrophysiology; Ca^2+^ imaging; 5-HT biosensors; afferent recordings	EC cells	5-HT_3_R-sensitive primary afferent fibers; mucosal sensory pathways	EC cells defined as polymodal chemosensors coupled to afferents	Pain-relevant afferent modulation
Kaelberer et al. [22]	2018	Mouse tissue; organoid–nodose coculture; optogenetics; iGluSnFR	Neuropod cells	Vagal nodose neurons; gut–brainstem sensory pathway	Rapid glutamatergic epithelial-to-vagal signaling	None—foundational sensory biology
Alcaino et al. [57]	2018	Mouse epithelial cultures/organoids; force stimulation; Piezo2 manipulation	Piezo2-positive EC/EEC subset	Neural pathway not directly tested	Piezo2 converts epithelial force into 5-HT release	Indirect/conceptual
Makadia et al. [63]	2018	Vil-ChR2 mice; epithelial optogenetics; afferent recordings; VMR assays	Broad villin-positive colonic epithelium	Extrinsic pelvic colon afferents; visceromotor reflex circuitry	Epithelial activation is sufficient to evoke afferent firing and VMR	Pain-relevant afferent modulation
Buchanan et al. [41]	2022	Duodenal stimulation; vagal recordings; CCK-lineage manipulation; behavior	CCK-labeled duodenal neuropod cells	Vagal nodose sensory pathway	Sugar vs. sweetener discrimination via transmitter-selective output	None—foundational sensory biology
Barton et al. [10]	2022	Mouse/human tissue; epithelial–DRG coculture; Gucy2c loss-of-function; linaclotide assays	GUCY2C^high^ neuropod-like epithelial cells	DRG sensory neurons; visceral nociceptive pathways	Neuropod-cell GUCY2C restrains DRG excitability and nociceptive signaling	Direct experimental visceral hypersensitivity
Bayrer et al. [64]	2023	Gut–nerve preparations; DRG Ca^2+^ imaging; VMR assays; EC-cell silencing/activation	EC cells	Mucosal afferents; lumbosacral DRG sensory neurons; visceral hypersensitivity circuitry	EC cells are necessary and sufficient for visceral hypersensitivity	Direct experimental visceral hypersensitivity
Alcaino et al. [58]	2025	Human duodenal organoids; *TPH1* reporter; electrophysiology; Ca^2+^/cAMP imaging; 5-HT assays	Human duodenal EC cells	Neural partner not directly tested	Human EC cells show multimodal sensory and secretory control	Indirect/conceptual

Abbreviations: EC, enterochromaffin cell; EEC, enteroendocrine cell; GC-C, guanylate cyclase C; cGMP, cyclic guanosine monophosphate; IBS-C, constipation-predominant irritable bowel syndrome; pERK, phosphorylated extracellular signal-regulated kinase; DRG, dorsal root ganglion; VMR, visceromotor response; 5-HT, serotonin/5-hydroxytryptamine; TPH1, tryptophan hydroxylase 1.

**Table 2:** Epithelial sensory cell populations and signaling programs relevant to gut neuroepithelial communication and visceral pain.

Epithelial Population	Defining Features/Markers	Input/Output	Neural Target (s)	Functional Role	Pain Relevance/Key Caveat
CCK-labeled nutrient-sensing neuropod cells [21,22,41]	CCK-lineage EEC sensory cells with basal neuropods and rapid synaptic coupling to vagal nodose neurons	Nutritive sugars and non-caloric sweeteners; glutamate for sugar signaling, purinergic output for sweetener-associated signaling	Vagal nodose neurons.	Rapid epithelial coding of luminal nutrient identity; sugar–sweetener discrimination	Conceptual relevance only; no direct pain evidence. Relationship to pain-regulatory neuropod-like cells remains unresolved
GUCY2C^high^ neuropod-like epithelial cells [10]	Rare GUCY2C^high^ epithelial cells with basal pseudopod-like projections, neuroendocrine identity, and synaptic gene enrichment	GUCY2C/GC-C agonism, including linaclotide; local pain-restraining epithelial signal, mediator unresolved	DRG sensory neurons/DRG-linked visceral afferent pathways.	Restraint of sensory-neuron hyperexcitability; preservation of linaclotide-responsive nociceptive suppression	Direct experimental pain relevance in mice. Mediator, communication mode, regional organization, and relationship to other neuropod subtypes remain unresolved
Murine enterochromaffin cells [23,56,57,64]	TPH1/5-HT-positive, electrically excitable epithelial sensory cells with chemosensory and mechanosensory programs	TRPA1 irritants, isovalerate, catecholaminergic signals, Piezo2-dependent force sensing; serotonin output	5-HT_3_R-sensitive mucosal sensory afferents; DRG-linked pain pathways in the Bayrer context.	Polymodal epithelial transduction; serotonergic afferent recruitment; amplification of afferent gain under pain-relevant conditions	Direct experimental pain relevance. EC cell–afferent communication mode, sex-dependent baseline states, and translational generalizability remain incompletely defined
Human enterochromaffin cells [58]	CRISPR-labeled TPH1-positive EC cells from human duodenal organoids	Bacterial metabolites, aromatic amino acids, adrenergic agonists, gut hormones; regulated serotonin release	Not directly established in pain-relevant human epithelial–neuronal systems.	Human multimodal EC-cell sensory integration and secretory control	Translational platform; human pain relevance unproven. Direct coupling to human sensory neurons and disease relevance in IBS remain unresolved
Broad villin-positive colonic epithelium in the Makadia model [63]	Villin-Cre–targeted ChR2-positive colonic epithelium; broad epithelial activation, subtype unresolved	Optogenetic epithelial activation; partially purinergic output consistent with ATP/UTP-sensitive afferent recruitment	Extrinsic pelvic colonic afferents.	Epithelial activation is sufficient to evoke afferent firing and visceromotor output	Direct nociception-relevant epithelial sufficiency; responsible epithelial subtype and full mediator profile remain unresolved

Abbreviations: CCK, cholecystokinin; EEC, enteroendocrine cell; EC, enterochromaffin cell; GUCY2C, guanylate cyclase C; GC-C, guanylate cyclase C; DRG, dorsal root ganglion; 5-HT, serotonin/5-hydroxytryptamine; 5-HT3R, 5-HT3 receptor; TRPA1, transient receptor potential ankyrin 1; Piezo2, Piezo-type mechanosensitive ion channel component 2; ATP, adenosine triphosphate; UTP, uridine triphosphate; IBS, irritable bowel syndrome.

**Table 3:** Levels of evidence supporting neuroepithelial regulation of visceral nociception.

Evidence Level	Biological Claim	Directly Demonstrated	Still Unresolved	Representative Studies
Level 1. Structural basis for neuroepithelial communication	Specialized epithelial sensory cells are anatomically organized for nerve-oriented signaling	Neuropod-bearing EECs show vesicle-rich basal projections, neuron-like cytoskeletal features, glial association, mucosal nerve contact, synaptic-associated machinery, and traceable epithelial–neuronal connectivity	Functional transmission speed, transmitter logic, and nociceptive relevance were not tested; anatomy alone does not prove pain-related signaling	[21,35]
Level 2. Functional epithelial-to-neural sensory transmission	Specialized epithelial cells can transmit biologically meaningful gut signals to neural pathways	Neuropod cells rapidly signal to vagal nodose neurons; CCK-labeled duodenal neuropod cells discriminate sugar from sweetener; EC cells release 5-HT and modulate serotonin-sensitive afferents	Vagal nutrient circuits are not spinal/DRG nociceptive circuits; chronic pain or persistent hypersensitivity was not directly demonstrated	[22,23,41]
Level 3. Epithelial activity is sufficient to recruit pain-relevant afferent/reflex output	Epithelial activation alone can evoke nociception-relevant afferent and reflex activity	Broad optogenetic activation of villin-positive colonic epithelium evokes extrinsic pelvic afferent firing and visceromotor responses; purinergic blockade reduces part of the response	Responsible epithelial subtype, full mediator profile, and relevance to chronic hypersensitivity remain unresolved	[63]
Level 4. Defined epithelial sensory lineages causally regulate visceral hypersensitivity	Specific epithelial sensory lineages can amplify or restrain pain-related signaling	EC-cell signaling recruits DRG activity, enhances VMRs, and is necessary/sufficient for experimental hypersensitivity; GUCY2C^high^ neuropod-like cells regulate DRG excitability and restrain nociceptive output	GUCY2C^high^ mediator and communication mode remain unknown; EC-cell disease relevance in human chronic pain disorders requires validation	[10,64]
Level 5. Clinically anchored epithelial analgesic relevance	Epithelial signaling pathways are therapeutically tractable and clinically concordant with abdominal pain modulation	Linaclotide reduces GC-C-dependent experimental hypersensitivity, suppresses colonic nociceptor firing, reduces dorsal horn pERK, and is clinically associated with abdominal pain improvement in IBS-C analyses	Human epithelial GC-C/GUCY2C analgesic mechanism is not fully proven; contribution of GUCY2C^high^ neuropod-like cells to human IBS-C pain relief remains untested	[10,75,76]

Abbreviations: EEC, enteroendocrine cell; EC, enterochromaffin cell; DRG, dorsal root ganglion; VMR, visceromotor response; GUCY2C, guanylate cyclase C; GC-C, guanylate cyclase C; pERK, phosphorylated extracellular signal-regulated kinase; IBS-C, constipation-predominant irritable bowel syndrome.

## Data Availability

No new data were created or analyzed in this study. Data sharing is not applicable to this article.
